# Unveiling the three-dimensional magnetic texture of skyrmion tubes

**DOI:** 10.1038/s41565-021-01031-x

**Published:** 2021-12-20

**Authors:** Daniel Wolf, Sebastian Schneider, Ulrich K. Rößler, András Kovács, Marcus Schmidt, Rafal E. Dunin-Borkowski, Bernd Büchner, Bernd Rellinghaus, Axel Lubk

**Affiliations:** 1 Leibniz Institute for Solid State and Materials Research, IFW Dresden, Dresden, Germany; 2 Dresden Center for Nanoanalysis, cfaed, Technische Universität Dresden, Dresden, Germany; 3 Ernst Ruska-Centre for Microscopy and Spectroscopy with Electrons and Peter Grünberg Institute, Forschungszentrum Jülich, Jülich, Germany; 4 Department Chemical Metal Science, Max Planck Institute for Chemical Physics of Solids, Dresden, Germany; 5 Institute of Solid State and Materials Physics, Technische Universität Dresden, Dresden, Germany; 6 Würzburg-Dresden Cluster of Excellence ct.qmat, Dresden, Germany

**Keywords:** Magnetic properties and materials, Magnetic devices, Imaging techniques

## Abstract

Magnetic skyrmions are stable topological solitons with complex non-coplanar spin structures. Their nanoscopic size and the low electric currents required to control their motion has opened a new field of research, skyrmionics, that aims for the usage of skyrmions as information carriers. Further advances in skyrmionics call for a thorough understanding of their three-dimensional (3D) spin texture, skyrmion–skyrmion interactions and the coupling to surfaces and interfaces, which crucially affect skyrmion stability and mobility. Here, we quantitatively reconstruct the 3D magnetic texture of Bloch skyrmions with sub-10-nanometre resolution using holographic vector-field electron tomography. The reconstructed textures reveal local deviations from a homogeneous Bloch character within the skyrmion tubes, details of the collapse of the skyrmion texture at surfaces and a correlated modulation of the skyrmion tubes in FeGe along their tube axes. Additionally, we confirm the fundamental principles of skyrmion formation through an evaluation of the 3D magnetic energy density across these magnetic solitons.

## Abstract (web summary)

Holographic vector-field electron tomography reveals the three-dimensional magnetic texture of Bloch skyrmion tubes in FeGe at nanometre resolution, including complex three-dimensional modulations and fundamental skyrmion formation principles.

## Main

As multidimensional solitons, skyrmions^1^ are localized in two dimensions, which requires a definite mechanism through additional frustrating magnetic couplings for their stabilization^2^ and application, for example, for advanced magnetic memories^3–5^. As a consequence of their solitonic character, they can condense into thermodynamically stable phases, in particular dense packed lattices under applied fields^1^. The stabilization mechanism of these phases and their formation principles are ruled by effective skyrmion–skyrmion interactions^2^. However, the morphology of these phases in the phase diagrams of real materials is dictated by the condensation physics of two-dimensional (2D) periodic arrays, as in vortex-lattices of type-II superconductors^1^. In particular, the field-temperature phase diagram may hold various transitions between different condensed phases of skyrmions^6,7^. Very recently, some studies addressed this problem for skyrmionic phases theoretically^8^ and experimentally^9^. In three-dimensional (3D) bulk materials or thicker films, the skyrmions are extended string-like objects; in the simplest formation they are homogeneously continued as skyrmion tubes (SkTs) preserving translational invariance along their axis. In magnetic nanoobjects, however, the influence of surfaces will affect formation, shape and interaction of skyrmions and the stabilization of condensed skyrmionic phases^10–15^. Already from the earliest observations of skyrmionic phases in films of chiral helimagnets^16,17^, it is known that their phase diagrams massively deviate from those of bulk materials. 3D surface twists can stabilize SkTs in thin films^10,11,18^ and 3D modulations of SkTs embedded in a conical host phase may introduce an attractive interaction between these tubes^19^. 3D SkT modulations also affect emergent electric and magnetic fields acting on spin-polarized electrons and magnons^20^, which results in unusual transport phenomena^21^ on top of the normal topological Hall effect in static and current-driven skyrmion crystals^22–25^.

Similarly, size, energy and coupling of the SkTs can be modified by their 3D modulations at the interface, for example, in hybrid chiral ferromagnet–superconductor systems^26^. Finally, the observation of unusually strong topological quantum Hall effects^27^ may indicate the presence of abrupt magnetization changes such as in Bloch points attached to magnetic bobbers in surface regions^28^.

Notwithstanding the importance of 3D effects, neither exact 3D models of SkTs in realistic confined geometries nor high-resolution experimental mappings of their spin texture are currently available, although effects of confinement^10,17^ and anisotropies^29^ have received attention. This lack of data prevents a deeper understanding of skyrmion lattice defects^15,30^, influence of surface anisotropies, curvatures^31^ and real structure effects in the modulation of 3D skyrmionic spin textures. Among the various high-resolution magnetic imaging techniques, transmission electron microscopy (TEM) based electron holography^32,33^ and X-ray magnetic chiral dichrosim^34–36^ can be conducted in a tomographic way to determine the 3D magnetic induction, **B**, or magnetization, **M**, of a sample, respectively. In this work, we use holographic vector-field electron tomography (VFET)^33^. It provides a substantially higher spatial resolution (below 10 nm) than X-ray based methods, which is crucial for resolving the details of magnetic textures in nanomagnetic structures such as vortices^33^ or skyrmions. The limited space in a high-resolution TEM instrument, however, has so far prevented any in situ applications of rotatable (out-of-plane) magnetic fields to a cryogenically cooled sample, which is essential for the acquisition of tomographic tilt series of electron holograms from a sample that needs to be magnetically stabilized. This limitation impedes the measurement and 3D reconstruction of spin textures for a large class of materials with a metastable skyrmion phase at non-zero applied fields below room temperature (for example, many isotropic helimagnets). For the present experiments, we have therefore devised a setup that overcomes these obstacles. To provide the crucial link between the experimentally obtained high-resolution **B** field data and the magnetization texture **M**, we use micromagnetic models.

## Vector-field tomography in an external magnetic field

**Fig. 1:**
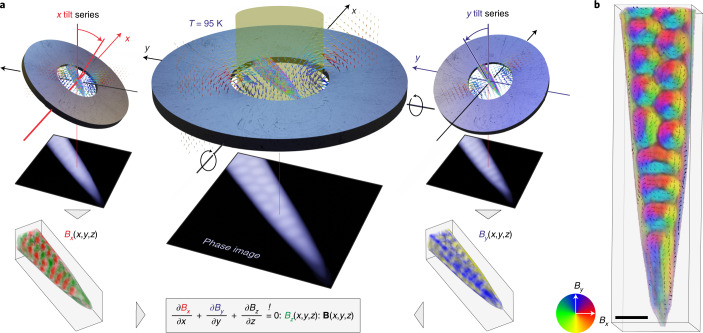
Holographic VFET of skyrmions in FeGe. **a**, Scheme of the VFET concept. A needle-shaped sample is placed above an out-of-plane magnetized Sm_2_Co_17_ ring in a liquid nitrogen cooled TEM holder. The low temperature and the remanent stray field of the ring stabilizes SkTs and their orientation with respect to the holder. A tilt series of 2D phase images of the transmitted electron wave is obtained from off-axis electron holographic tilt series around the *x* (left) and *y* axes (right). Subsequently, *x* and *y* components of the magnetic induction **B** are tomographically reconstructed from the corresponding phase tilt series. Solving ${{{\rm{div}}}}\,{{{{{\mathbf{B}}}}}}=0$ finally yields the *z* component, hence the full 3D vector of **B**(*x*, *y*, *z*). **b**, 3D map of the resulting magnetic induction. For clarity, only the experimental *B*_*x*_ and *B*_*y*_ components are shown here. Scale bar, 100 nm.

**Extended Data Fig. 1:**
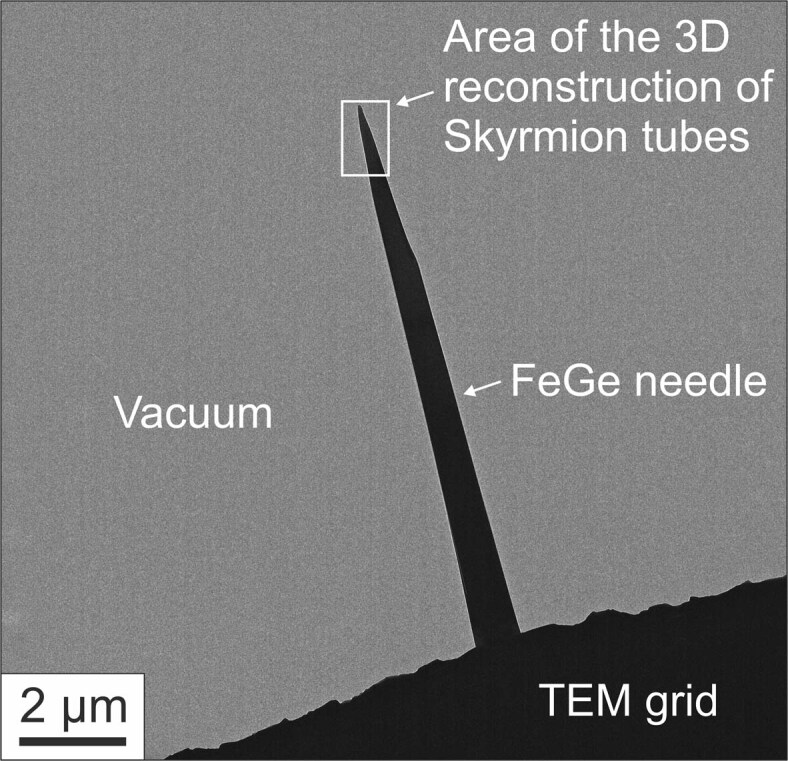
Needle-shaped FeGe sample. Bright-field TEM image of the FeGe specimen used for the holographic VFET experiment. The box indicates the area investigated by off-axis EH.

The tomographic investigation of the magnetic texture of skyrmions was conducted on a sample of the isotropic helimagnet FeGe with *P*2_1_3 structure (B20 phase). The material was chosen, since FeGe is an otherwise well-studied archetypical skyrmion host with a rather large skyrmion phase pocket in the phase diagram spanned by temperature and external field^16,37^. A needle-shaped sample (Extended Data Fig. 1 and Supplementary Information) was cut from a FeGe single crystal by focused ion beam (FIB) including ion polishing to restrict the ion beam damage to a surface layer of some nanometres (Methods and Supplementary Information). The dimensions and shape of the needle ensure that, even at high tilt angles, the sample is fully electron transparent and the obtained holographic projections cover the same sample region. Additionally, the elongated shape has some technological significance for anticipated spintronic devices such as racetrack memories^38^. To (1) adjust the skyrmion phase below the Curie temperature and (2) stabilize the orientation of the skyrmion lattice with respect to the TEM holder, the FeGe needle was steadily exposed to an out-of-plane magnetic field of *μ*_0_*H*_ext_ ≈ 170 mT. The field was provided through the remanent stray field of a ring-shaped Sm_2_Co_17_ hard magnet that was placed under the sample in a tomography-adapted liquid nitrogen TEM cooling holder (Fig. 1a). The field is virtually homogeneous across the micrometre-sized sample (Supplementary Information).

Using this special setup, we have recorded three holographic tilt series as required for VFET^33^ (see Methods and Supplementary Information for details of the imaging conditions). The first series of holograms was acquired by tilting the sample around the *x* axis at room temperature, since above the Curie temperature of *T*_C_ = 278.7 K (ref. ^39^), the phase *φ*_e_ reconstructed from the holograms is of pure electrostatic origin. The (scalar) electrostatic potential *Φ* was then determined by inverting the Radon transformation (that is, linear projection) linking *Φ* and *φ*_e_. The resulting 3D mean inner potential distribution is nearly homogeneous as discussed in Supplementary Information (see Methods for the tomographic reconstruction details).

In the following two series, the sample was tilted around the *x* and *y* axes (Fig. 1a) at *T* = 95 K. At this temperature below *T*_C_, the magnetic fields impose an additional Aharonov–Bohm phase *φ*_m_ on the imaging electrons. After subtracting the predetermined electrostatic contribution from the total phase shift, the in-plane components of the magnetic induction, *B*_*x*_(*x*, *y*, *z*) and *B*_*y*_(*x*, *y*, *z*) (Fig. 1b), were reconstructed from the remaining *φ*_m_ in 3D by inverse Radon transformation of another linear projection law linking the gradient of *φ*_m_ and *B*_*x*,*y*_ (see Methods for details). The spatial resolution of the reconstructed *B*_*x*_ and *B*_*y*_ components was better than 10 nm in directions outside the missing tilt range (Supplementary Information).

To change the tilt axis from *x* to *y*, the sample required to be warmed up to room temperature, rotated in-plane by 90° in the sample holder and field-cooled again. As a result, at the here-investigated and most confined tip region of the FeGe needle, the skyrmion patterns obtained after cooling before acquiring the *x* and *y* tilt series were not altered. On the contrary the pattern in the less confined broader end of the needle was changed (see Supplementary Fig. 1 for details).

On the basis of *B*_*x*,*y*_, the remaining third component *B*_*z*_(*x*, *y*, *z*) was determined by solving ${{{\rm{div}}}}\ {{{{{\mathbf{B}}}}}}=0$, thereby yielding the full 3D vector-field of the magnetic induction **B**(*x*, *y*, *z*) (see Methods for details and Supplementary Video 1 for 3D animations of the tomograms). We finally note that tomographic reconstruction is (mildly) ill-conditioned, hence it requires regularization to mitigate the unavoidable reconstruction error at the expense of spatial resolution^40^. Since the strength of the regularization (for example, width of a low-pass filter) is not well-determined, we therefore tested different regularization strengths before picking the best trade-off between noise and resolution for further analysis (Supplementary Information). Note that this procedure is further complicated because of the different noise amplification in all three Cartesian components of **B**, reconstructed from different datasets with different missing wedges. Subsequently, we only discuss magnetization features, which are discernible at all tested regularizations.

## Magnetic texture of SkTs

**Fig. 2:**
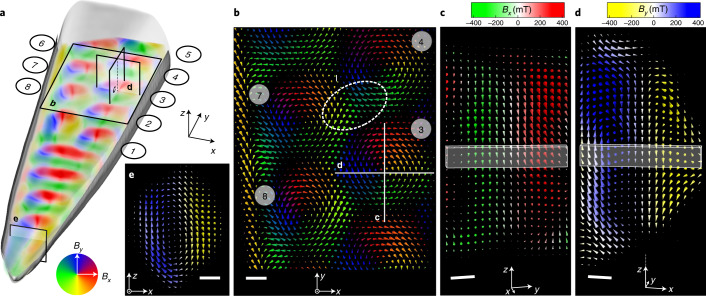
Spin texture of SkTs in a FeGe needle. **a**, Volume rendering (coloured) of (*x*, *y*) components of the reconstructed magnetic induction **B** and iso-surface of the mean inner potential visualizing the sample shape (grey, bottom half only). **b**–**e**, Cross-sections in the *xy* plane (**b**), *yz* plane through SkT 3 (**c**), *xz* plane through SkT 3 (**d**) and *xz* plane through the tip of the needle (**e**) at positions indicated by rectangles in **a**. The size of the arrows depicts the magnitude of the total magnetic induction, whereas the colour coding shows the magnitude of the corresponding out-of-plane component. For the cross-sections through SkT 3 (**c** and **d**), the SkT was artificially aligned along its *z* axis. A 17-nm-thick surface layer and the stray field were excluded, since these areas contain artefacts from the sample preparation and the 3D imaging (Supplementary Information). Scale bars, 20 nm.

In the following, we analyse this comprehensive 3D set of **B**(*x*, *y*, *z*) data to extract characteristic magnetic features and quantities of the SkTs in FeGe. Herein, we focus on Bloch-like features, which intrinsically have small magnetic charges and hence allow for a straight forward interpretation of magnetization in terms of **B** (see Supplementary Information for details). Figures 1b and 2a,e reveal that the tip of the needle hosts a single longitudinal vortex (vortex line pointing along *y*), which eventually transforms to a single SkT by pushing the vortex line upward into the *z* direction. It follows a row of three SkTs that are elliptically distorted towards the sideward surfaces of the needle, that is, perpendicular to both the needle axis and the stabilizing external field. Towards the broader back of the needle (top region of Fig. 1b), these elongated SkTs develop into a zig-zag chain of Bloch SkTs, when the width surpasses a critical value of roughly 150 nm. This width corresponds to about twice the characteristic helical modulation length *L*_D_ and the next-nearest neighbour distance in a close-packed skyrmion lattice in FeGe^30^. An evaluation of the out-of-plane component of **B** (Supplementary Information) reveals a ratio of areas with positive and negative *B*_*z*_ of 1.5, which also points to a close-packing of the SkTs in this region^8^.

**Fig. 3:**
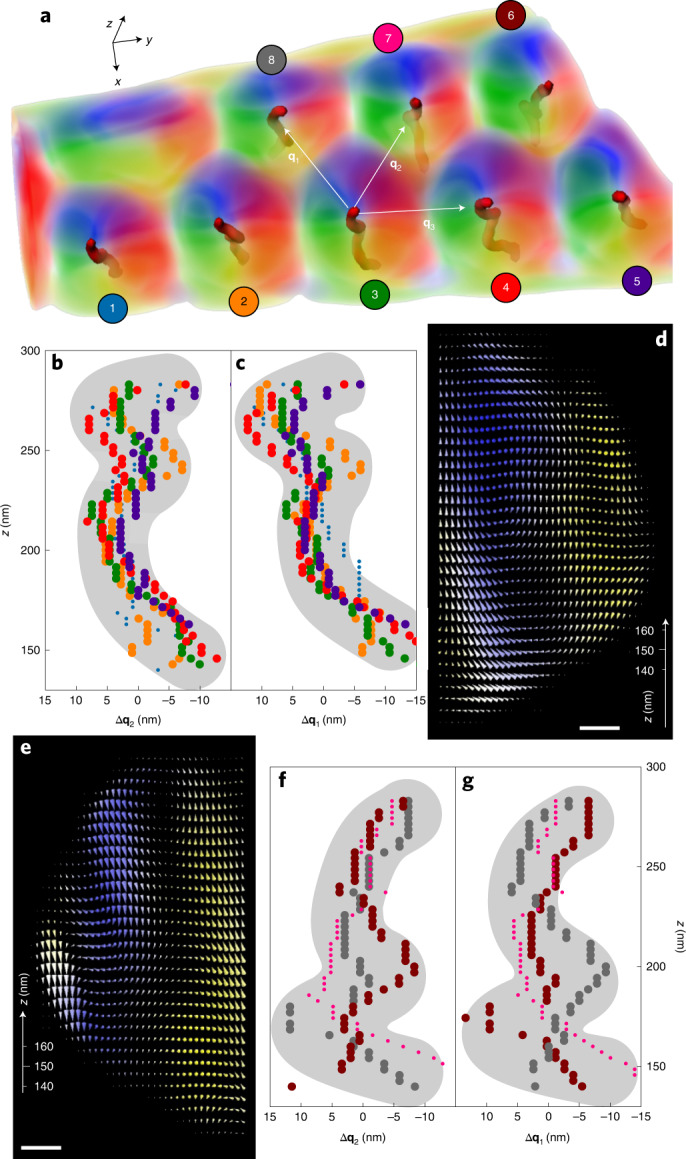
Axial modulations of the SkTs. **a**, 3D rendering of the (*x*, *y*) components of **B** for eight close-packed SkTs in the FeGe needle. The red lines represent the SkT axes, and **q**_1_, **q**_2_, **q**_3_ indicate the three nearest neighbour directions of the SkT lattice. **b**,**c**, *z* dependent positions of the cores of SkTs 1–5 along **q**_2_ (**b**) and **q**_1_ (**c**) as well as SkTs 6–8 (**f**,**g**) along **q**_2_ (**f**) and **q**_1_ (**g**). The data points are coloured according to the labels in **a**. **d**,**e**, *xz* slices through SkTs 3 (**d**) and 8 (**e**). Scale bars, 20 nm.

Figure 2 and Supplementary Videos 2–4 represent an in-depth view of the **B** field within these SkTs. Figure 2b shows a planar cross-section of **B** through the vertical centre of the needle. Here, the colour of the arrows indicates the direction of the in-plane (*x*, *y*) components of **B** according to the colour wheel in Fig. 2a. While all four SkTs in this section feature radial Bloch walls, details of the **B** field exhibit subtle discrepancies from that of an undisturbed, perfect Bloch skyrmion. (1) The skyrmions may exhibit significant distortions and (partially) lose their axial symmetry. Neither the direction of the in-plane components of **B** remain tangential nor is its magnitude constant for a given radius. (2) Unlike expected for isolated magnetic solitons, some distortions of the skyrmionic spin textures are accompanied by magnetic flux leaking between neighbouring SkTs as highlighted by the dashed region ‘I’ between SkTs 4 and 7. The similarity of this texture with a confined helical band suggests the evolution of a metastable isolated skyrmion towards a helical modulation (strip-out) discussed in bilayer thin films^41^. To gain further insight into these buried features, Figs. 2c,d show two orthogonal vertical slices through SkT 3. Since the SkT axes are found to be bent and partially twisted (below and Fig. 3), SkT 3 was artificially aligned along the *z* axis for this presentation. To this end, each *xy* slice of the tube was laterally shifted such that the minima of the in-plane component ${\mathbf{B}}_{\perp }=\sqrt{{B}_{x}^{2}+{B}_{y}^{2}}$ of all slices are aligned along the *z* axis. Both cross-sections confirm the lack of axial symmetry and substantiate the overall inhomogeneity of the magnetic texture in the SkT already seen from the planar cross-section. In contrast to pure Bloch SkT, we observe small contributions of radial Néel-type modulations in the local magnetic induction. These imperfections grow on approaching the surface and finally lead to a total collapse of the skyrmion structure. This becomes most apparent in the *xz* cross-section in Fig. 2d, where the thickness of the needle decreases. This region should be understood as result of surface symmetry breaking and concomitant effects, such as pinning by surface anisotropies, modified magnetic properties due to FIB surface damage and demagnetization fields. Finally, at the very tip a longitudinal vortex state emerges (Fig. 2e), reducing the demagnetizing field. Subsequently, the vortex core bends, ultimately forming a transverse SkT.

**Extended Data Fig. 2:**
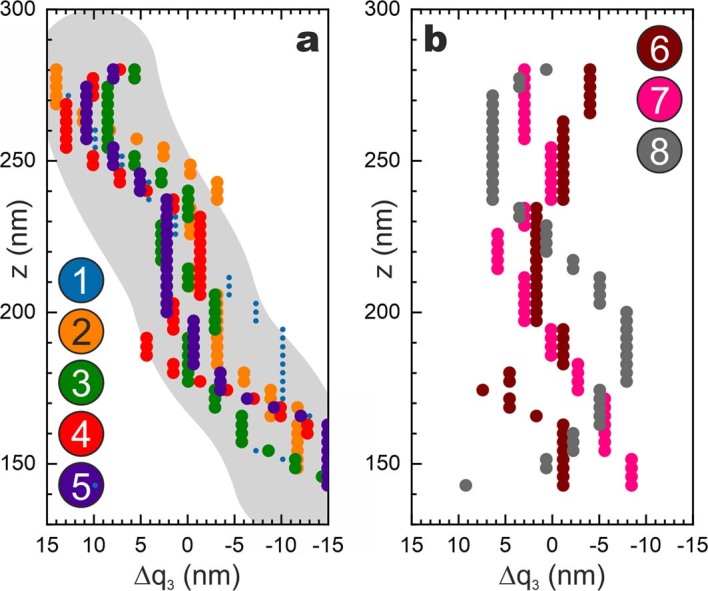
Axial modulations of the SkTs. **a** and **b**, *z* dependent positions of the cores of SkTs 1-5 and SkTs 6–8 along **q**_3_. The data points are coloured according to the labels in Fig. 4a.

In the cubic helimagnet FeGe, a twisting in the third direction, that is along the axis of the SkT, could result in a gain of energy through the Dzyaloshinskii–Moryia (DM) exchange. However, such an effect will not create triply twisted structures of skyrmions, because the ferromagnetic vector can only rotate in two directions in the cutting plane perpendicular to the skyrmion axis. Hence, the chiral twist^10^ could only affect the shape of the SkTs. For example, a modulation could arise as a tertiary conformational deviation from straight cylindrical SkT shape^42,43^. Confinement or surface pinning may promote such morphology changes. Indeed, Fig. 3a illustrates that the axis of SkTs (red lines) are axially bent and twisted rather than extending as straight cylindrical objects along the *z* axis in the close-packed region of the needle (similar rendering of **B**_⊥_ as in Fig. 2a). To study possible correlations between these deformations, we have analysed the in-plane positions of the SkT axes along the nearest neighbour directions **q**_1_, **q**_2_, **q**_3_ (indicated by white arrows). The resulting dependencies of the deviations from an average axial position along **q**_2_ and **q**_1_, that is, in directions that are largely affected by the lateral confinement, are shown in Fig. 3b,c for the bottom row of SkTs (nos. 1–5) and in Fig. 3f,g for SkTs 6–8 in the top row. Except for SkT 1 (small blue circles in Fig. 3b,c), which is least closely packed and has two elliptically elongated SkT neighbours, and SkT 7 (small pink circles in Fig. 3f,g), which is additionally distorted due to an unusual magnetic coupling to SkT 4 (above), all SkT axes exhibit pronounced sideward deformations. As indicated by grey bands (guides to the eye only), these lateral modulations are correlated among SkTs in the same row. They have a modulation length of approximately 80 nm that is close to the helical period *L*_*D*_ ≃ 70 nm in FeGe^16^ pointing to the DM interaction as a possible origin of the deformations. Note, however, that comparisons with *yz* cross-sections through SkTs 3 and 8 in Figs. 3d,e reveal that these modulations correlate with the occurrence of edge states^44^. These edge states reside at the sidelong rims of the FeGe needle (see left and right surfaces in Figs. 1b and 2a) and are separated by very narrow magnetic transition regions (resembling domain walls) of some 10 nm in width from the SkTs. The correlation of the deformation of the SkTs with these edge states is corroborated by the facts that (1) the central deformations are directed towards the centre on both sides of the needle and (2) the magnetic orientations of the edge states and the outer rims of the SkTs’ spin textures are concurrently reversed between the right (SkTs 1–5) and left side (SkTs 6–8) of the needle, respectively. This results in qualitatively identical interactions between the SkTs and the edge states on either side. In contrast, the deformations of the SkTs along the largely unconfined direction **q**_3_ do not exhibit strong correlations beyond a tilt of the first SkT row (Extended Data Fig. 2).

## Magnetic energy density distribution

The 3D **B** field data allows us to experimentally derive from the volume of a sample spatial maps of free energy density contributions from magnetic exchange and DM interactions, respectively. These energetic contributions are most essential for the formation and stabilization of skyrmions and SkTs, as they are expected to reduce the free energy in the centres of the SkTs, while the regions of in-plane magnetization regions in a SkT lattice may be considered as domain walls of increased energy^45^. We have calculated from **B**(*x*, *y*, *z*) the solenoidal part of the magnetic exchange energy density

$$\mathit{w}_\mathrm{ex}=\frac{A}{\mu_0^2 M_{\rm{s}}^2}\left|(\nabla \times \mathbf{B}) \right|^2$$

and the volume contribution of the DM energy density

$${{w}}_{{{{\rm{DM}}}}}=\frac{D}{{\mu }_{0}^{2}{M}_{\rm{s}}^{2}}{{{{{\mathbf{B}}}}}}\cdot (\nabla \times {{{{{\mathbf{B}}}}}}).$$

**Fig. 4:**
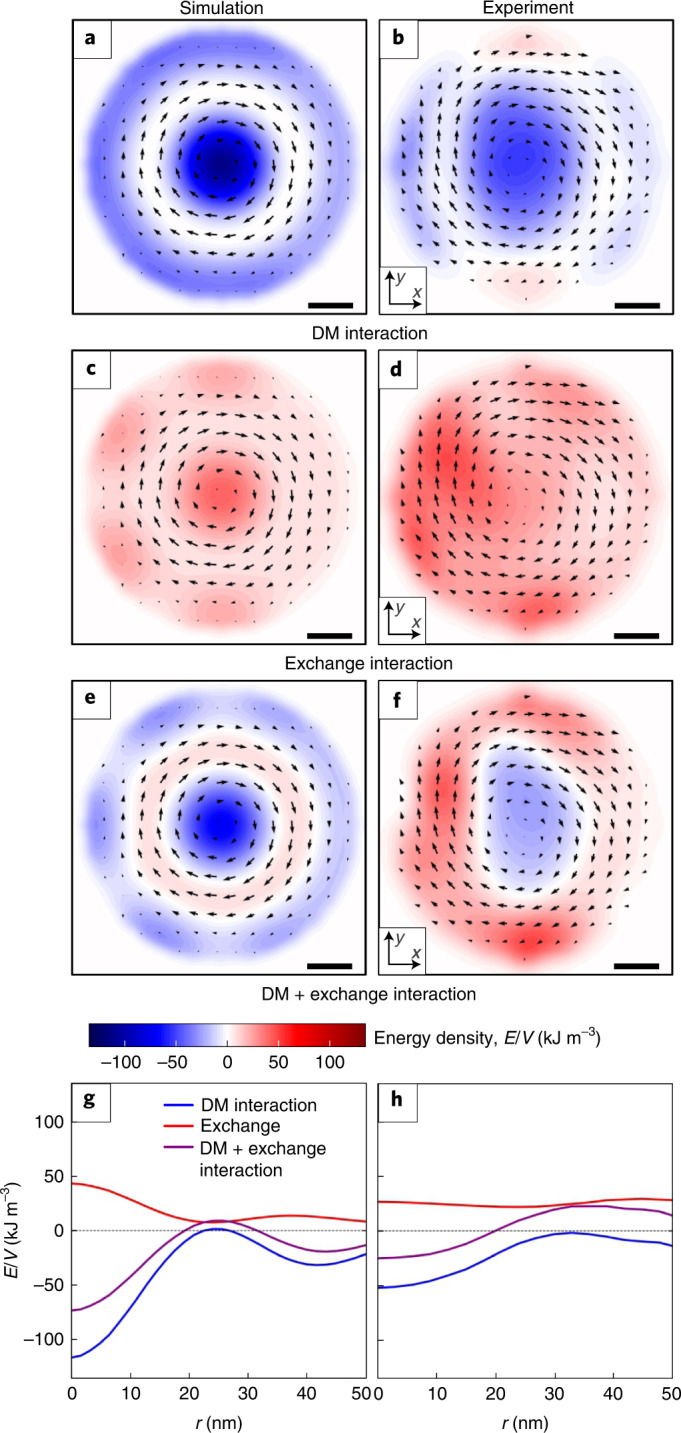
Comparison of simulated and experimental planar maps of the predominant magnetic energy density contributions. **a**,**b**, DM interaction $\frac{D}{{\mu }_{0}^{2}{M}_{\rm{s}}^{2}}{B}_{z}{(\nabla \times {{{{{\mathbf{B}}}}}})}_{z}$ derived from the simulation (**a**) and the experiment (**b**). **c**,**d**, Exchange interaction $\frac{A}{{\mu }_{0}^{2}{M}_{\rm{s}}^{2}}{\left|{\left(\nabla \times {{{{{\mathbf{B}}}}}}\right)}_{z}\right|}^{2}$﻿ derived from the simulation (**c**) and the experiment (**d**). **e**,**f**, Sum of DM and exchange﻿ interaction derived from the simulation (**e**) and the experiment (**f**). **g**,**h**, Rotational averages of **e** and **f** as a function of the radius (*r*)﻿ derived from the simulation (**g**) and the experiment (**h**). Overlaid arrow plots in **a**–**f** indicate the in-plane magnetic induction. Scale bars, 15 nm.

Here, $A=8.78\,{{{{\rm{pJ}}}}}\,{{{{\rm{m}^{-1}}}}}$ and $D=1.58\,{{{{\rm{mJ}}}}}\,{{{{{\rm{m}}}}}^{-2}}$ denote the exchange stiffness and the DM interaction strength, respectively, and ${M}_{\rm{s}}=384\,{{{{\rm{kA}}}}}\,{{{{\rm{m}^{-1}}}}}$ the saturation magnetization of FeGe^46^. Due to the vanishing magnetic charge density *ρ*_m_ ≈ 0, the conservative part of the exchange energy ${\left|\nabla \cdot {{{{{\mathbf{M}}}}}}\right|}^{2}$ is small in Bloch skyrmions. Other contributions are total derivatives that can be collapsed to surface terms, and are therefore neglected. As the spin texture of the SkTs is highly disturbed in the near-surface region (Fig. 2c,d), and to account for the axial deformation of the SkTs, only magnetic induction data from the central part of the SkT (see grey shaded boxes in Figs. 2c,d) was used and projected in the *xy* plane to calculate the planar distribution of energy densities. For comparison, such energy density maps were also calculated for a simplified skyrmion lattice model using the circular cell approximation^47^, taking into account the shape of the needle (Supplementary Information). Figure 4 shows the resulting simulated and experimentally determined energy density maps for the contributions arising from the DM and exchange interactions, and their sum. Here, we only plot energy densities dominated by the in-plane components of the magnetic induction to suppress some of the artefacts afflicting the *B*_*z*_ component, which is, however, sufficient and consistent with calculations that take the full **M**(*x*, *y*, *z*) into account (Supplementary Information). Accordingly, the experimental results agree well with the simulation with respect to the overall distribution of energy minima and maxima, but also show deviations concerning absolute values and size of the SkTs. In particular, the course of the rotationally averaged contribution (Fig. 4g,h) confirms experimentally the prediction that the reduction in the free energy density due to the DM interaction overcompensates the energetic costs of the exchange in the core of the SkT. Note, furthermore, that the reconstructed energy densities show an overall positive net energy of the SkT structure. Due to the missing exchange and DM interaction terms, surface and bulk anisotropies, Zeeman energy and previously discussed reconstruction errors, we, however, cannot conclude whether the positive total energy indicates a metastability of the Bloch SkTs (see also ref. ^10^ for theoretic discussion). In comparison with the simulations, the experimental energy density landscapes reveal slightly larger overall radii of the SkTs that appear furthermore slightly compressed in the *x* direction. While the former may be related to shortcomings of the circular cell model when approaching the interstitial regions between the SkTs the latter is attributed to the interaction of the SkT with the edge state and contributes, besides the noise, to the quantitative reduction of the rotational averages in Fig. 4h.

## Conclusions

We used low-temperature holographic VFET in combination with the spatial stabilization of the specimen’s magnetic state by an external magnetic field to reconstruct the full vector-field **B** of the skyrmionic spin texture in FeGe in all three dimensions at nanometre resolution. The unrivalled resolution of this 3D magnetic microscopy of a volume sample revealed detailed insights into the specifics of the 3D spin texture of skyrmions.

Besides a characterization of the complicated breakdown of the skyrmion texture on approaching the tip of the needle, for example, emergence of the longitudinal vortex state and the surfaces in axial directions, we observed a variety of real structure effects in the spatial extension of SkTs. Among them were axial and planar distortions of the SkTs, local losses of axial symmetry and the occurrence of unexpected radial rather than purely tangential tilts of the magnetic induction in the circumferential Bloch walls. Even indications of in-plane magnetic flux leaking among neighbouring SkTs in close-packed regions were found. Also, the 3D course of the SkT axes was investigated in great detail. Here, we observed a substantial bending and twisting of these axes that was locally correlated with the occurrence of pronounced edge states, specifically in directions that were affected by confinements. Noteworthy, these deformations appeared at length scales, where harmonic modulations were promoted by the DM interaction. The energy maps across the SkTs confirmed experimentally the anticipated formation and stabilization mechanism of skyrmions by a frustrated interplay of different exchange energies.

We anticipate further improvement of VFET in terms of spatial resolution and reconstruction quality by integration of in situ vector magnets as well as three-tilt axis tomography holders, thereby removing persisting ambiguities in the analysis of spin textures and energy densities in a wide array of complex magnetic textures, including other members of the skyrmion family.

## Methods

### Sample preparation

On the basis of the results of crystal growth by chemical vapour transport in the system Fe/Ge^48^ single crystals of FeGe in the B20 structure were grown via chemical transport reaction using iodine as a transport agent. Starting from a homogeneous mixture of the element powders iron (Alfa Aesar 99.995%) and germanium (Alfa Aesar 99.999%) the cubic modification of FeGe crystallized by a chemical transport reaction very slowly in a temperature gradient from 850 K (source) to 810 K (sink) and a transport agent concentration of 0.2 mg cm^−3^ iodine (Alfa Aesar 99.998%). The chemical vapour transport was made perpendicular to the tube axis over a diffusion distance of 38 mm. Selected crystals were characterized by EDXS, WDXS and especially X-ray single crystal diffraction to verify the present modification.

The preparation of the FeGe needle was carried out via FIB technique on a Thermo Fisher Scientific Helios 660 operated at 30 kV. A rough cut of the needle geometry (700 × 700 nm^2^) was performed with currents of 790 and 430 pA. For further fine shaping (300 × 300 nm^2^) the current was reduced to 80 and 40 pA. The final polishing was carried out at 24 pA. To remove preparation residue, the needle was finally cleaned in a Fischione Model 1070 NanoClean for 1 min.

### Acquisition and reconstruction of the holographic tilt series

Holographic tilt series were recorded at an FEI Titan G2 60-300 HOLO in Lorentz-Mode (conventional objective lens switched off) operated at 300 kV. The voltage of the electrostatic Möllenstedt bisprism was set to 120 V leading to a fringe spacing of 2.3 nm in the electron hologram (Supplementary Information). For the acquisition of the latter, a GATAN K2 Summit direct detection camera in counting mode was used yielding a holographic fringe contrast of 40%. The acquisition process was performed semi-automatically with an in-house developed software package^49^ to collect three holographic tilt series consisting of object and object-free empty holograms, two at 95 K and one at room temperature. For the first tilt series at 95 K, the angle between the needle and tilt axis amounted to 30°. For the second tilt series, the specimen was manually rotated outside the microscope in-plane by 70° (ideal is 90°) resulting in an angle between the needle and tilt axis of −40° (Supplementary Information for the details). The tilt range of each tilt series was from −66° to + 65° in 3° steps. To obtain the full phase shift (>2π), the phase images were unwrapped automatically by the Flynn algorithm and manually at regions, where the phase signal was too noisy or undersampled, by using previous knowledge of the phase shift (for example, from adjacent projections)^40^. Potential phase wedges in vacuum caused by the magnetic stray field of the ring were corrected in all three-tilt series. An analysis of these stray-field contributions is presented in the Supplementary Information.

### Tomographic reconstruction

All three phase image tilt series were aligned, that is, corrected for image displacements with respect to their common tilt axis by cross-correlation, centre-of-mass method and common-line approach^33^. The thereby obtained aligned datasets correspond to the following linear projection laws (Radon transformations):

1 $${\varphi }_{{\mathrm{e}}}\left(p,\theta ,z\right)={C}_{{\mathrm{E}}}{\iint }_{{{{{{\mathbf{e}}}}}}\cdot {{{{{\mathbf{r}}}}}}}{{\varPhi }}\left(x,y,z\right){{{\rm{d}}}}x{{{\rm{d}}}}y$$

and

2 $$\frac{\partial {\varphi }_{{\mathrm{m}}}(p,\theta ,z)}{\partial z}=\frac{e}{\hslash }{\iint }_{{{{{{\mathbf{e}}}}}}\cdot {{{{{\mathbf{r}}}}}}}{B}_{p = y,x}\left(x,y,z\right){{{\rm{d}}}}x{{{\rm{d}}}}y.$$

Here, *C*_E_ is a kinetic constant depending solely on the acceleration voltage, *p* and *z* are the 2D detector coordinates, *θ* the tilt angle, **r** = (*x*, *y*)^*T*^ and ${{{{{\mathbf{e}}}}}}={(\cos \theta ,\sin \theta )}^{T}$. The index to the integral indicates a collapse of the 2D integral to the projection line defined by **e** ⋅ **r**. The subsequent tomographic 3D reconstruction of the aligned phase tilt series (that is, the inverse Radon transformation) was numerically carried out using weighted simultaneous iterative reconstruction technique (W-SIRT)^50^.

The three resulting tomograms represent the incremental 3D phase shift per voxel that we refer to as 3D phase maps. The two 3D phase maps obtained at 95 K were released from their electrostatic contribution by superposition and subtraction of the 3D phase map obtained at room temperature. Then, the derivation of each of the two resulting magnetic 3D phase maps in directions perpendicular to both the experimental tilt axis and tilt directions using an appropriate Fourier filter (Fourier-slice theorem) as well as multiplication with the factor *ℏ*/*e* leads to one component of the magnetic induction in the respective direction. Since the specimen was rotated only by 70° in the underlying tomographic experiment for the reconstruction of these two **B** field components, one of them was projected on the orthogonal direction of the other to receive finally the 3D *B*_*x*_ and *B*_*y*_ components. 3D visualization was performed using the Avizo software package (ThermoFisher Company) and the Mayavi Python package. A verification of the experimental workflow repeated on simulated data is provided in Supplementary Information.

### Calculation of the third magnetic B field component

The third **B** field component *B*_*z*_ is obtained by solving Gauss’s law for magnetism ${{{\rm{div}}}}\ {{{{{\mathbf{B}}}}}}=0$ with appropriate boundary conditions on the surface of the reconstruction volume. Here, we used periodic boundary conditions for solving this differential equation in Fourier space endowed with coordinates **k**, that is,

3 $${B}_{z}\left({{{\bf{k}}}}\right)=-\frac{{k}_{x}{B}_{x}\left({{{\bf{k}}}}\right)+{k}_{y}{B}_{y}\left({{{\bf{k}}}}\right)}{{k}_{z}}$$

The zero frequency component (integration constant) was fixed by setting the average of *B*_*z*_ to zero on the boundary of the reconstruction volume. To suppress noise amplification by this procedure, a Butterworth-type low-pass filter was applied.

### Magnetic energy densities

Following ref. ^33^ the exchange energy density may be split into contributions from magnetic charges, currents and surface terms

$$\frac{A}{{M}_{\rm{s}}^{2}}\left({\left(\nabla \cdot {{{{{\mathbf{M}}}}}}\right)}^{2}+{\left|\nabla \times {{{{{\mathbf{M}}}}}}\right|}^{2}\right)-{{w}}_{{{{\rm{surf}}}}}.$$

In the magnetostatic limit considered here, the magnetization in the second term may be replaced by **B**/*μ*_0_ and can be reconstructed from the tomographic data. In the case of the DM interaction, we have the following identities

4 $$\begin{array}{ll}{E}_{{{\mbox{DM}}}}\left[{{{{{\mathbf{M}}}}}}\right]&=\frac{D}{{M}_{\rm{s}}^{2}}\int {{{{{\mathbf{M}}}}}}\cdot \left(\nabla \times {{{{{\mathbf{M}}}}}}\right){{{\rm{d}}}}V\\ &=\frac{D}{{\mu }_{0}^{2}{M}_{\rm{s}}^{2}}\int {{{{{\mathbf{B}}}}}}\cdot \left(\nabla \times {{{{{\mathbf{B}}}}}}\right){{{\rm{d}}}}V+\frac{D}{{M}_{\rm{s}}^{2}}\int \nabla \cdot \left({{\varPhi }}{{{{{{\mathbf{j}}}}}}}_{{\mathrm {b}}}\right){{{\rm{d}}}}V\\ &=\frac{D}{{\mu }_{0}^{2}{M}_{\rm{s}}^{2}}\int {{{{{\mathbf{B}}}}}}\cdot \left(\nabla \times {{{{{\mathbf{B}}}}}}\right){{{\rm{d}}}}V+\frac{D}{{M}_{\rm{s}}^{2}}\oint\!\!\!\int {{{{{{\mathbf{w}}}}}}}_{{{{\rm{surf}}}}}\cdot {{{\rm{d}}}}{{{{{\mathbf{S}}}}}}\end{array}$$

Here **j**_b_ denotes the bound current and *Φ* the scalar magnetic potential. The last line identifies that part of the DM energy density, which can be derived solely from the **B** field, and may be identified as a volume contribution, which can be reconstructed from tomographic data. The remainder can be collapsed to a surface term.

## Online content

Any methods, additional references, Nature Research reporting summaries, source data, extended data, supplementary information, acknowledgements, peer review information; details of author contributions and competing interests; and statements of data and code availability are available at 10.1038/s41565-021-01031-x.

## Supplementary information

Supplementary Information Supplementary Figs. 1–14 and Discussion.

Supplementary Video 1 Circulating magnetic induction **B** of a skyrmion lattice in a FeGe needle. On the left side, the animation shows the volume rendering of the reconstructed out-of-plane component *B*_*z*_ with the colour coding red (+0.4 T), white (0 T) and blue (−0.4 T). In the right panel, the superimposed volume renderings of the reconstructed in-plane components *B*_*x*_ (horizontal) with colour coding red (+0.3 T), white (0 T), green (−0.3 T) and *B*_*y*_ (vertical) with colour coding blue (+0.3 T), white (0 T), yellow (−0.3 T) is displayed. The size of the specimen is approximately 240 nm in the thickest part (top). The animation sequence is as follows: time action, 0–8 s, rotation of both volumes around the vertical axis by 360°. 8–10 s, rotation of the *B*_*z*_ component (left) around the vertical axis by −80° circle side view of *B*_*z*_. 10–12 s, cutting of the volumes from the side (position indicated by a red slice). 12–14 s, reverse cutting with rotation of *B*_*z*_ around the vertical axis by 80°. 14–16 s, cutting of both volumes from the front. 16–18 s, rotation of both volumes around the vertical axis by −90° circle cut side view. 18–20 s, reverse slicing from right to left and rotation (back) around the vertical axis by a 90° circle. The initial orientation of the two volumes is reached.

Supplementary Video 2 *Z* slicing of the magnetic induction **B** of a skyrmion lattice in a FeGe needle. In the upper panel, the animation shows the volume rendering of the *B*_*x*_ component from the side (*yz* view). In this representation, the SkTs are visualized as green–red pairs. The lower panel displays *z* slices from the top (*xy* view) of the reconstructed in-plane components as arrow plots, as well as *B*_*x*_ (vertical) with colour coding red (+0.2 T), white (0 T), green (−0.2 T) and *B*_*y*_ (horizontal) with colour coding blue (+0.2 T), white (0 T), yellow (−0.2 T). The *z* slices are simultaneously shown in the upper panel to illustrate their position.

Supplementary Video 3 Slicing of the magnetic induction **B** of a skyrmion lattice in a FeGe needle. On the left side, the animations show arrow plots taken at slices parallel to the nearest neighbour direction *q*_1_ (see main text). The positions of the slices are indicated as black rectangles on the right, where the superimposed volume renderings of the reconstructed in-plane components *B*_*x*_ (horizontal) with colour coding red (+0.3 T), white (0 T), green (−0.3 T) and *B*_*y*_ (vertical) with colour coding blue (+0.3 T), white (0 T), yellow (−0.3 T) are visualized. The colour coding of the arrows is consistent with the colours in the volume rendering (colour wheel).

Supplementary Video 4 Slicing of the magnetic induction **B** of a skyrmion lattice in a FeGe needle. On the left side, the animations show arrow plots taken at slices parallel to the nearest neighbour direction *q*_2_ (see main text). The positions of the slices are indicated as black rectangles on the right, where the superimposed volume renderings of the reconstructed in-plane components *B*_*x*_ (horizontal) with colour coding red (+0.3 T), white (0 T), green (−0.3 T) and *B*_*y*_ (vertical) with colour coding blue (+0.3 T), white (0 T), yellow (−0.3 T) are visualized. The colour coding of the arrows is consistent with the colours in the volume rendering (colour wheel).

## Data Availability

The data that support the findings of this study are available from the corresponding author on reasonable request.

## Extended data

are available for this paper at 10.1038/s41565-021-01031-x.

## Code availability

Software codes that support the findings of this study are available from the corresponding author on reasonable request.
