# Supplementary material for: Unveiling the three-dimensional magnetic texture of skyrmion tubes
Source: Nat Nanotechnol. 2021 Dec 20;17(3):250–5. doi: 10.1038/s41565-021-01031-x (PMC8930765; doi:10.1038/s41565-021-01031-x)
Supplement: Supplementary file 1 — Supplementary Figs. 1–14 and Discussion. [file 41565_2021_1031_MOESM1_ESM.pdf]

nature portfolio

https://doi.org/10.1038/s41565-021-01031-x

Supplementary information

Unveiling the three-dimensional magnetic texture of skyrmion tubes

In the format provided by the
authors and unedited

Supplementary Information

Daniel Wolf[1,†], Sebastian Schneider[2, 1,†], Ulrich K. Rößler[1], András Kovács[3], Marcus Schmidt[4], Rafal E. Dunin-Borkowski[3], Bernd Büchner[1,5,6], Bernd Rellinghaus[2], and Axel Lubk[1,5,6,*]

[1]Leibniz Institute for Solid State and Materials Research, IFW Dresden, Helmholtzstr. 20, 01069 Dresden, Germany
[2]Dresden Center for Nanoanalysis, cfaed, Technische Universität Dresden, 01069 Dresden, Germany
[3]Ernst Ruska-Centre for Microscopy and Spectroscopy with Electrons and Peter Grünberg Institute, Forschungszentrum Jülich, 52425 Jülich, Germany
[4]Department Chemical Metal Science, Max Planck Institute for Chemical Physics of Solids, Nöthnitzer Str. 40, 01187 Dresden, Germany
[5]Institute of Solid State and Materials Physics, TU Dresden, 01069 Dresden, Germany
[6]Würzburg-Dresden Cluster of Excellence ct.qmat, Germany
[†]These authors contributed equally to this work.
[*]Corresponding author.

September 29, 2021

SI 1 Specimen and tilt geometry

To perform the holographic VFET experiment at cryogenic conditions, we investigated a needle-shaped specimen prepared by focused ion beam (FIB) milling from a FeGe single crystal and transferred it to a lift-out TEM grid. Fig. S1 shows the needle geometry and positions on the sample, where the tilt series were recorded (Fig. S1a). Moreover, Figs. S1b, c illustrate the orientation of the sample with respect to the tilt axis and the skyrmionic lattice arrangement for tilt series 1, 3, and Figs. S1d, e for tilt series 5, 6 after 70° clock-wise in-plane rotation of the sample outside the TEM instrument at room temperature. Before rotation, two additional tilt series (2 and 4 in Figs. S1b, c) were acquired at room temperature (RT) and reconstructed in order to subtract their electrostatic 3D phase maps from the ones obtained at 95 K. Unfortunately, warming up the sample to room temperature, rotating the sample outside the microscope, and cooling down again to 95 K in the microscope led to a slight modification of the skyrmion lattice in the thicker region, clearly visible in Fig. S1c and Fig. S1e. Consequently, these data could not be used for 3D magnetic vector field reconstruction.

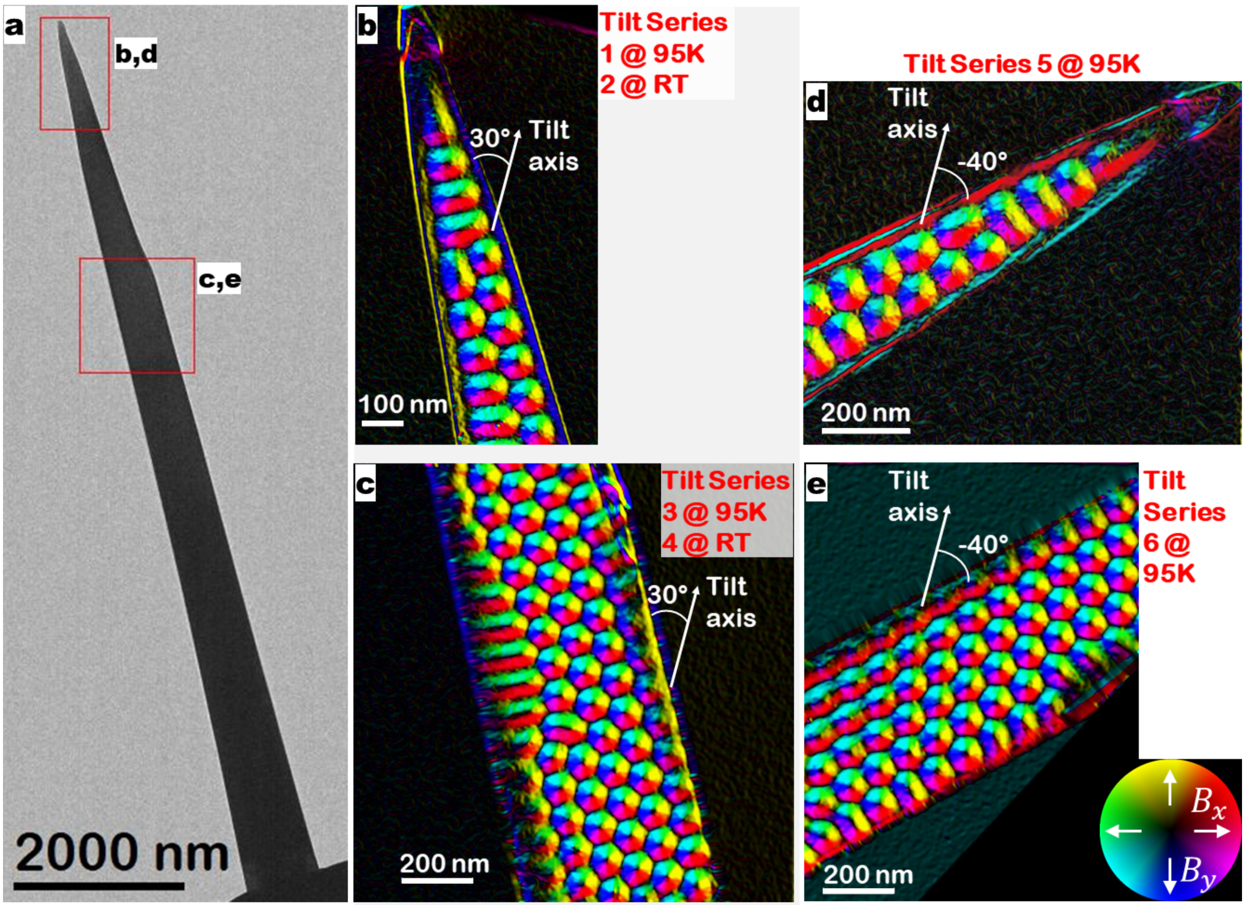

Figure S1: FeGe specimen and tilt geometry of the holographic VFET experiment. **a**, Bright-field TEM image of the ca. 10 µm long free-standing needle-shaped specimen. **b-e**, Reconstructed projected magnetic in-plane $\boldsymbol{B}$ field (color-wheel maps indicating the x and y direction of $\boldsymbol{B}$) at 0° tilt angle and positions indicated in **a**.

SI 2 Surface modification

Unlike homogeneous bulk materials, the investigated needle is nanoscopically small with a large surface area-to-volume ratio. As a consequence, the magnetic texture can be easily modified in these surface regions influencing the structure of the whole skyrmion. The effect of the sample preparation by focussed ion beam (FIB) milling was investigated, since the highly energetic ions are partially implanted in the surface area of the needle, and may create defect cascades and even lead to partial amorphization. Hence, to understand the effect of FIB-structuring, the defect landscape of the cross-section of an equivalent FeGe FIB lamella was characterised with HRTEM and EDX mapping. After preparation of a conventional FIB FeGe sample, the lamella was coated with amorphous carbon. Subsequently, a thin slap was cut out of the sample to investigate the cross-section of the lamella.

HRTEM measurements (see Fig. S2a) reveal that the inner part of the sample is still fully crystalline. This core, however, is surrounded by a roughly 4 nm thin shell, which is marked by red dotted lines. Complementary STEM-EDX investigations show that there is still iron present in this amorphous layer (see Fig. S2b), but no germanium (see Fig. S2c). Instead a strong oxygen signal can be found at the surface (see Fig. S2d). Furthermore a strong gallium signal can be found, which reaches up to 17 nm into the lamella volume in this sample area (see Fig. S2e). Thus we conclude that Ga^+ ions are implanted into the lamella during the FIB milling and preferentially removed germanium from the sample surface. The unbound iron gets oxidized and forms an amorphous layer.

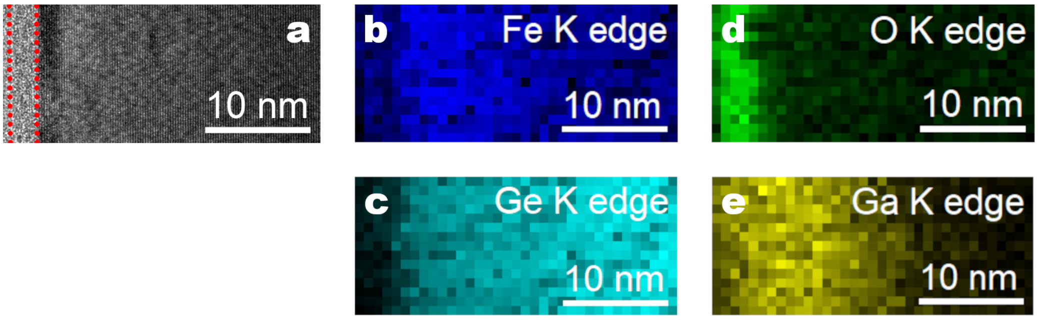

Figure S2: HRTEM and STEM-EDX investigations of the crystalline structure and elemental composition of the surface of a FeGe lamella prepared by FIB. **a**, The HRTEM measurement shows an amorphous layer (marked by red dotted lines) on top of the crystalline core. **b-e**, Complementary STEM-EDX investigations reveal, that this amorphous layer is an iron oxide, which results from the removal of Ge by the Ga^+ ions during FIB procedure.

Since the investigated FeGe needle most likely also features such an amorphous surface, and due to the fact that artifacts stemming from imaging aberrations, contamination during the tilt series acquisition and alignment errors may become pronounced at the surface regions, we excluded a surface layer of 17 nm for the 3D analysis of the $\boldsymbol{B}$ field tomogram. This is shown in Fig. S3 in which the volume rendering of B_x and B_y (colorwheel color-coding) and the MIP tomogram (grey) are superimposed.

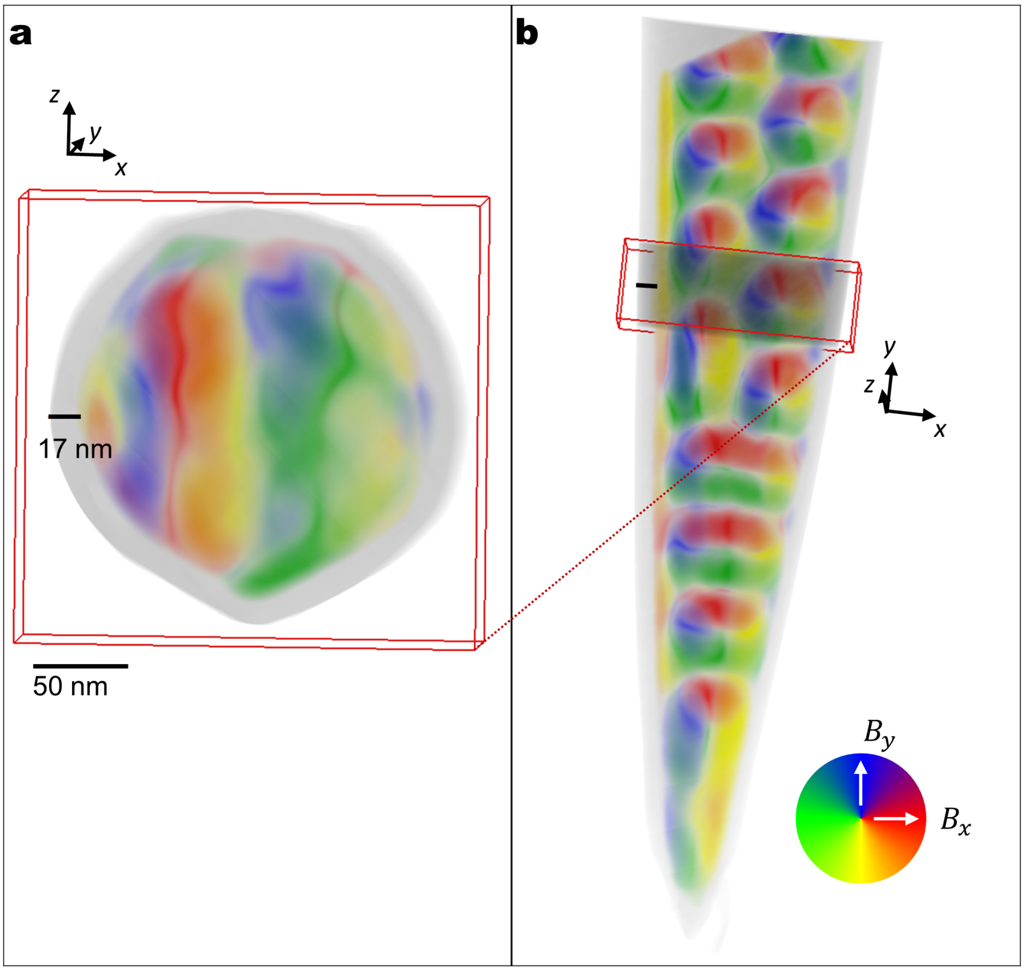

Figure S3: Visualization of the surface layer that was excluded for the 3D analysis of the $\boldsymbol{B}$ field tomogram of the needle-shaped FeGe sample. **a**, Cross-section view of a sub-volume extracted from **b**, in which the volume rendering of B_x and B_y (colorwheel color-coding) and the MIP tomogram (grey) are superimposed. The grey layer illustrates the surface layer not taken into account for our $\boldsymbol{B}$ field evaluation.

SI 3 Magnetic ring

The skyrmion phase within the needle was stabilized by the stray field of a ring-shaped rare earth permanent magnet fitted into a GATAN 636 double tilt nitrogen cooling TEM holder. Sm_2Co_{17} was chosen for the ring material as it offers a high out-of-plane saturation magnetization, high Curie point, good temperature stability and oxidation resistance [1]. The magnetic ring was prepared from a commercial cylindrical bulk magnet (outside radius: 1.5 mm, height: 1.5 mm) by sinker spark eroding and mechanical grinding to possess a hole with an inside radius of 150 µm and a thickness of 50 µm (see photograph in Fig. S4b). The oxidation resistance is crucial during the preparation process, as only the surface of the Sm_2Co_{17} gets oxidized, whereby the magnetic performance of the ring is only slightly degraded [2].

In order to test the properties of the ring after it's fabrication procedure, the magnetic induction was determined by magnetostatic simulations using the above-mentioned geometry and a value of the saturation magnetization of 1.1 T oriented in out-of-plane direction. The resulting B_z component is shown in Fig. S4a. The red-blue contrast reveals that the stray-field within the hole points in the opposite direction of the magnetization as also illustrated by the black field lines. A line profile taken at the lower surface of the ring (Fig. S4c) confirms that the applied external field in out-of-plane direction is 170 mT over a region of few tens of µm in the center of the hole sufficient to stabilize the skyrmion phase in the FeGe specimen.

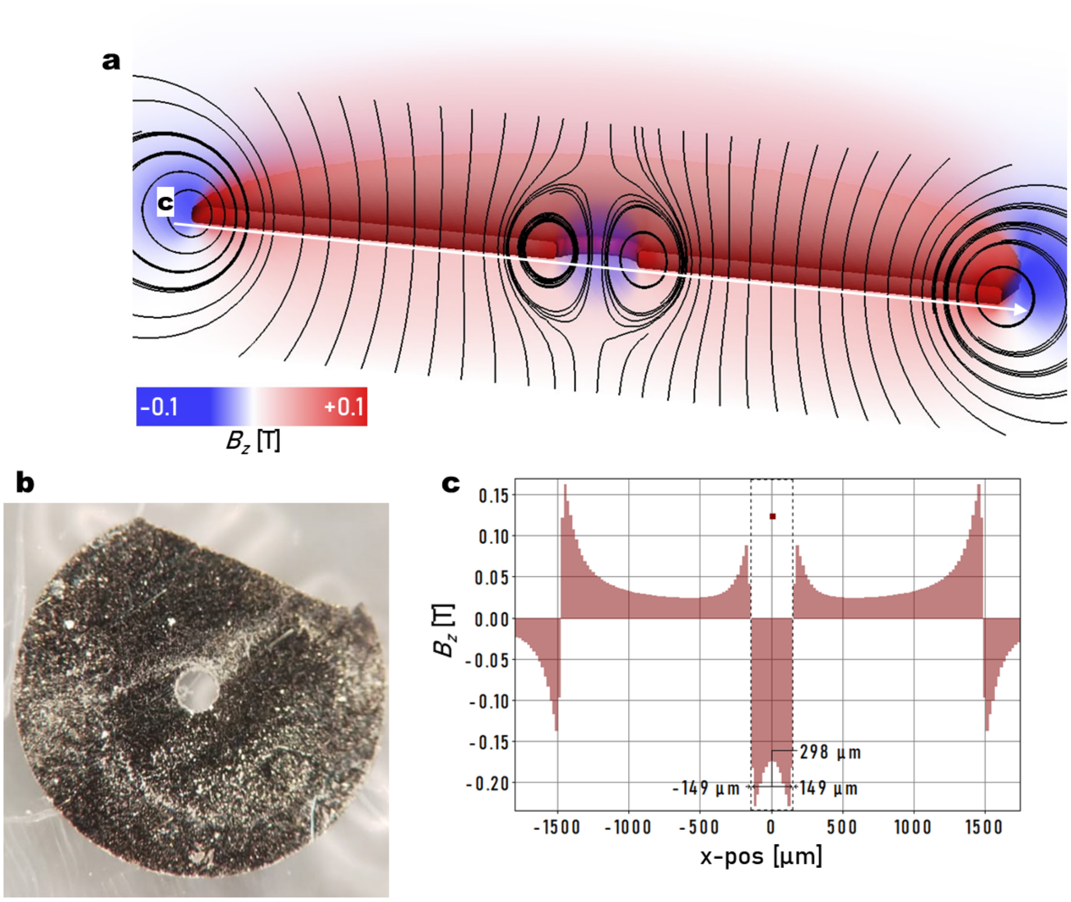

Figure S4: Sm_2Co_{17} permanent hard magnetic ring (50 µm thickness with outer and inner diameter of 3 mm and 300 µm) used for application of an external out-of-plane field on the FeGe specimen. **a**, Volume rendering (blue-white-red) of the B_z component of the ring (cut through the center) obtained by magnetostatic simulations with black field-lines illustrating a $\boldsymbol{B}$ field slice through the center of the ring. **b**, Photograph of the ring after usage in the TEM. **c**, Line profile of the B_z component taken at the surface of the ring at the position indicated by a white arrow in **a** (i.e., where the FeGe sample was placed in the experiment).

SI 4 3D electrostatic mean inner potential (MIP)

A 3D reconstruction of the FeGe needle-shaped FIB prepared sample at room temperature was carried out, in order to obtain the electrostatic contribution to the phase shift at each tilt angle

$$\varphi_{el}(x,y) = C_{\mathrm{E}} \int V_0(x,y,z)\,dz \tag{1}$$

that is proportional (interaction constant for 300 kV electrons is $C_{\mathrm{E}} = 0.0065\,\frac{\mathrm{rad}}{\mathrm{Vnm}}$) to the 3D electrostatic potential, i.e., mainly the mean inner potential (MIP). The MIP is of particular interest for quantitative 2D electron holography, to determine the thickness of the sample[3]. As described in the methods section of the main text, the tilt series were recorded from $-66°$ to $+65°$, hence the reconstructed tomogram suffers from missing wedge artefacts due to the incomplete tilt range ($\pm 90°$). These missing wedge artefacts in Fourier space reduce the resolution in the directions not covered in the projection data and hamper an accurate measurement of the MIP. For this reason, we applied a so-called finite support approach for missing wedge correction at low spatial frequencies in the tomogram[4], prior to the 3D analysis of the potential data. The 3D electrostatic potential analysis (see Fig S5) yields a MIP of 20.2 eV.

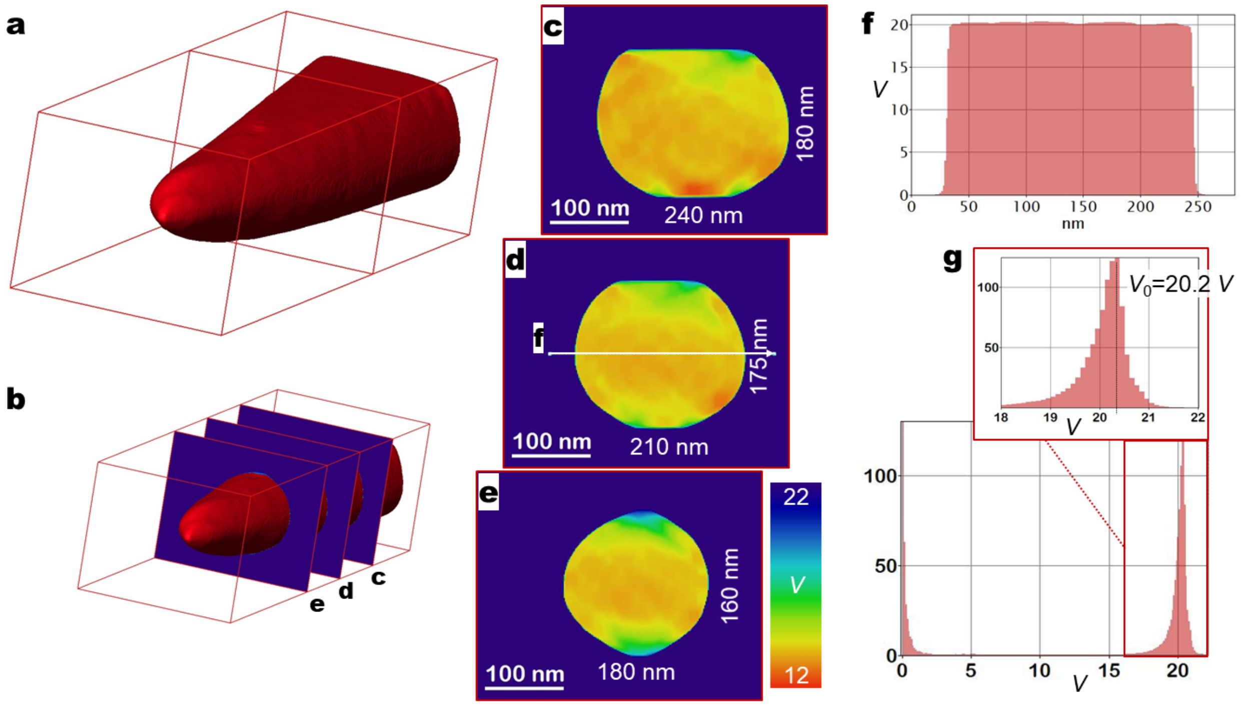

Figure S5: 3D electrostatic potential of the FeGe needle-shaped FIB-prepared sample reconstructed by electron holographic tomography at room temperature. **a**, 3D Iso-potential surface (15 V) visualizing the overall morphology. **b**, Same as a but with slices included to display three cross-sections, **c-e**, from bottom to the top of the sample. **f**, Potential profile along the line scan indicated as arrow in **d** revealing a plateau of 20 V that is consistent with a peak in the histogram **g** of the FeGe sample at 20.2 V.

SI 5 Spatial resolution of tomographic reconstruction

VFET involves a complex reconstruction process and data treatment from the raw data (tilt series) band-limited in both projection space (detector sampling) and angular space (finite tilt steps, limited tilt range) to the final tomograms. This data treatment includes, e.g., interpolation, Fourier and spatial filtering, as well as regularization within the tomographic reconstruction algorithm. Therefore, it is not possible to describe the signal and noise propagation in a straightforward manner, and to provide an overall isotropic lateral resolution value for the obtained tomograms. Here, we therefore provide an experimental resolution measure obtained from the sharpness of the object edge in the MIP tomogram (Fig. S6). Fig. S6a shows an x-z slice of the reconstructed MIP tomogram of the FeGe FIB needle, Fig. S6b its Fourier transform (FT). In the latter, the superposition of central slices (the FT of the projections) is clearly visible as well as the missing wedge in vertical direction beyond the experimental tilt range. The red dashed circle illustrates the band, in which spatial frequencies until 0.1 nm^{-1} are transferred corresponding to a 10 nm resolution in real space. A line profile is depicted in Fig. S6c along the line scan through the cross-section in Fig. S6a to obtain the edge spread functions on both sides of the object. Its width is determined by computing the derivative in direction of the line scan, e.g. in x-direction, yielding the so-called line spread function. We assign the full width half maxima (FWHM) of the two peaks of 10 nm to an estimated value of the lateral resolution. In Ref.[5] we used Fourier shell correlation to assess the lateral resolution of similar reconstructed VFET data in a more elaborated way, obtaining a value of 7 nm.

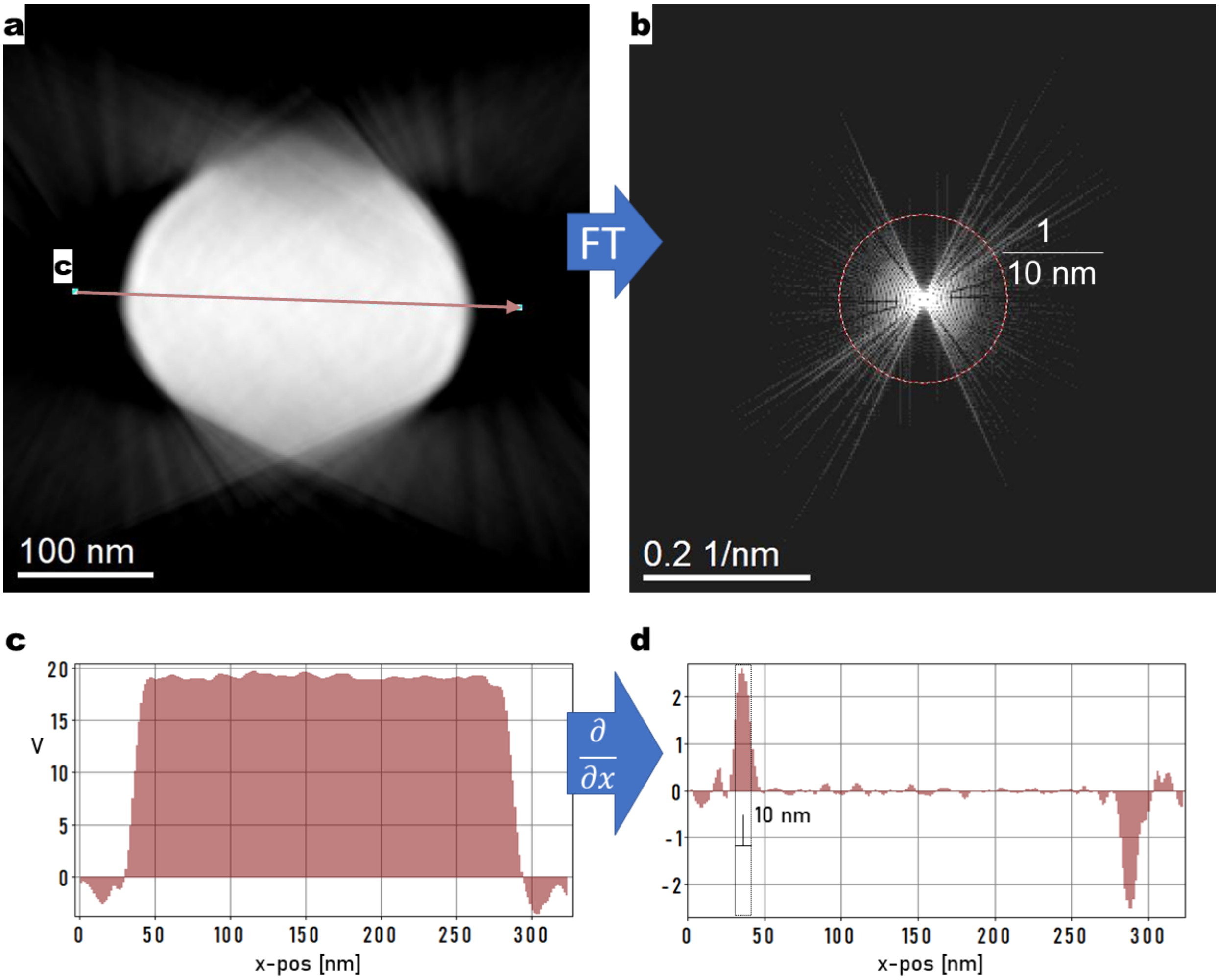

Figure S6: Spatial Resolution of tomographic reconstruction. **a**, Representative slice of the MIP tomogram showing the cross-section of the FeGe needle-shaped FIB sample. **b**, Fourier transform of **a**. **c**, Line profile along the line scan in indicated be an arrow in **a**. **d**, Numerical derivative of **c**.

SI 6 Regularization of tomographic reconstruction

Tomographic reconstruction is a (mildly) ill-conditioned inverse problem, which inevitably leads to an dramatic amplificaion of the input error (due to non-projective artifacts such as detection noise) in the output, when seeking an optimal reconstruction in terms of Euclidean error metric (square distance) between the original data and the projections of the reconstructed data. This problem is further amplified by incomplete set of tilt angles ("missing wedge", see SI 5) leaving whole spatial frequency domains unsampled, long-ranging electric and magnetic fields reaching outside of the the reconstructed field of view, which violate the support theorem of tomography, and the different impact of the above issues on the three Cartesian components of $\boldsymbol{B}$ in VFET. Therefore, every tomographic reconstruction incorporates some regularization scheme, which dampens the reconstruction error at the expense of some regularization error and reduction of spatial resolution. In (VF)ET, the regularization strength (e.g., width of the low-pass filter, iteration number) cannot be determined uniquely, because the input error (i.e., the deviation from the recorded "projections" from true projections of a vector field) is not well known in advance for three main reasons:

1. Detector noise in terms of a 4D noise transfer function is not well known.

2. Several unknown non-projective artifacts are introduced in the recording (e.g., dynamical diffraction), preprocessing (e.g., alignment errors, tilt angle error), and holographic reconstruction (e.g., phase unwrapping artefacts).

3. x- and y-components of $\boldsymbol{B}$ are affected by different missing wedges and input errors as they are derived from different tilt series. B_z inherits their reconstruction errors in the process of solving $\mathrm{div}\boldsymbol{B} = 0$.

It is therefore virtually impossible to (I) determine an optimal regularization strength, and (II) to compute the error by error propagation.

Considering these fundamental problems, we adopted the following phenomenological strategy to asses the reconstruction error. We performed several tomographic reconstructions employing different regularization schemes, compared the results and picked the best balance between reconstruction error (e.g., local fluctuation of $\boldsymbol{B}$ field) and spatial resolution (e.g., sharpness of edges) for further analysis and publication. We discuss only those local magnetic field features in the paper, which are discernible in different regularizations (e.g., SkT modulation). In Fig. S7, a comparison of three regularization schemes is depicted.

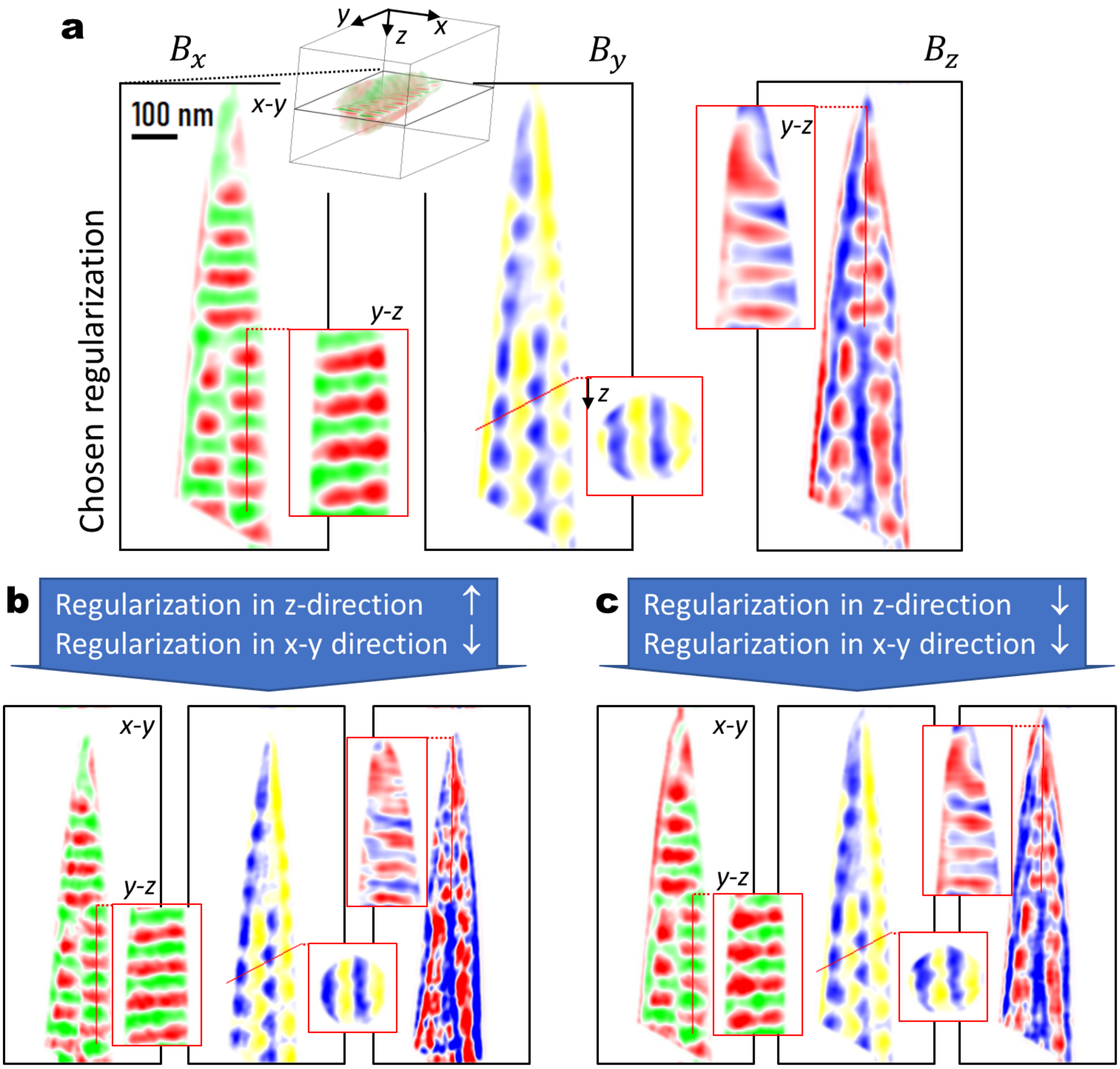

Figure S7: Comparison of three 3D reconstructions with different tomographic regularization schemes. **a**, Slices through 3D Magnetic induction maps (B_x, B_y, B_z), which we have identified to have the best balance between reconstruction errors and spatial resolution and hence chosen for the main text. **b**, same as **a** but with higher regularization in z direction and lower in x-y direction. Therefore, the SkTs are less modulated (y-z slice) in the B_x and B_y components, but the B_z component, which is calculated from B_x and B_y, is more noisy. **c**, same as **a** but with lower regularization in z direction. Therefore, the SkTs are more modulated (y-z slice).

SI 7 Micromagnetic modeling of skyrmion lattice

The elastic scattering mechanism of electrons within a magnetic field via minimal coupling renders VFET blind to the magnetic field strength $\boldsymbol{H}$. Consequently, VFET reconstructs only the solenoidal part of the magnetic texture $\boldsymbol{M} = \frac{1}{\mu_0}\boldsymbol{B} - \boldsymbol{H}$ in the magnetostatic limit considered here. In the (mostly 2D) literature, employing high-resolution TEM methods, this issue is tackled by comparing the (projected) magnetic induction with (micro)magnetic simulations, inferring the magnetization from a good fit of the corresponding $\boldsymbol{B}$ fields (see also Wolf et al.[5]). In the following we clarify to what extend $\boldsymbol{B}$ is sufficient to infer information about the magnetic texture of Bloch skyrmion tubes considered in this work.

To generate a theoretical magnetic model of the skyrmion lattice in the needle we carried out the following steps: (I) Synthesis of an artificial magnetic skyrmion texture by assembling a double line of SkTs with the help of the Circular Cell Approximation[6]. Herein we used the exchange constants $A = 8.78\ \frac{\mathrm{pJ}}{\mathrm{m}}$, $D = 1.58\ \frac{\mathrm{mJ}}{\mathrm{m}^2}$[7], an experimentally determined circular cell radius of 40 nm, and an external field of 170 mT as produced by the magnetic ring (Sec. SI 3). Dipolar interaction was taken into account through effective easy-plane anisotropy approximation in thin films[6]. The numerical solution of the non-linear differential equation as a boundary value problem pertaining to the polar angle of the magnetization in the circular cell was carried out with the help of the shooting method implemented in the DifferentialEquations package of Julia programming language. (II) In between the circular cells magnetization pointing into z direction along the external fields was assumed. Truncation to the outer shape of the nanowire was imposed by multiplication with a shape function of the needle, (III) Solution of the magnetostatic equations, $\Delta\Phi_M = \operatorname{div}\boldsymbol{M}$, $\boldsymbol{H} = -\nabla\,\Phi_M$, $\boldsymbol{B} = \mu_0\,(\boldsymbol{M} + \boldsymbol{H})$, within a supercell containing vacuum around the needle imposing periodic boundary conditions. The supercell (i.e., vacuum region) was chosen large enough to suppress boundary effects.

This idealized double-line SkT model of the needle subsequently served as the basis for evaluating the impact of the missing $\boldsymbol{H}$ information for the magnetization features discussed in the main text. Herein, we focus on the more subtle features, namely the Néel modulations (main text Fig. 2), SkT modulation (main text Fig. 3), and energy density profiles (main text Fig. 4 and SI 9). The broader features such as the longitudinal vortex in the tip, stripe-out of the skyrmions, and the boundary domain are produced by an underlying magnetization texture in a straightforward manner. We also employed the above model to generate artificial tomographic tilt series in SI 12.

We modeled the SkT modulation (main text Fig. 3) by shifting the skyrmion center as a harmonic function of z. Accordingly, the SkT modulation produces a negligible $\boldsymbol{H}$ field only and is therefore equally represented in both $\boldsymbol{M}$ and $\boldsymbol{B}$ (see Fig. S8a). As an exemplary Néel modulation we employed the chiral surface twist model, which gradually changes the character of the skyrmion from Bloch to Néel, when approaching the surface[8]. Such a modulation produces a strong $\boldsymbol{H}$ field (Néel modulations intrinsically harbor magnetic charges), which even dominates the $\boldsymbol{B}$ field (see Fig. S8b). Consequently, Néel modulations are also present in $\boldsymbol{B}$, however, the relation to $\boldsymbol{M}$ is not straightforward, even including a sign change in the considered example.

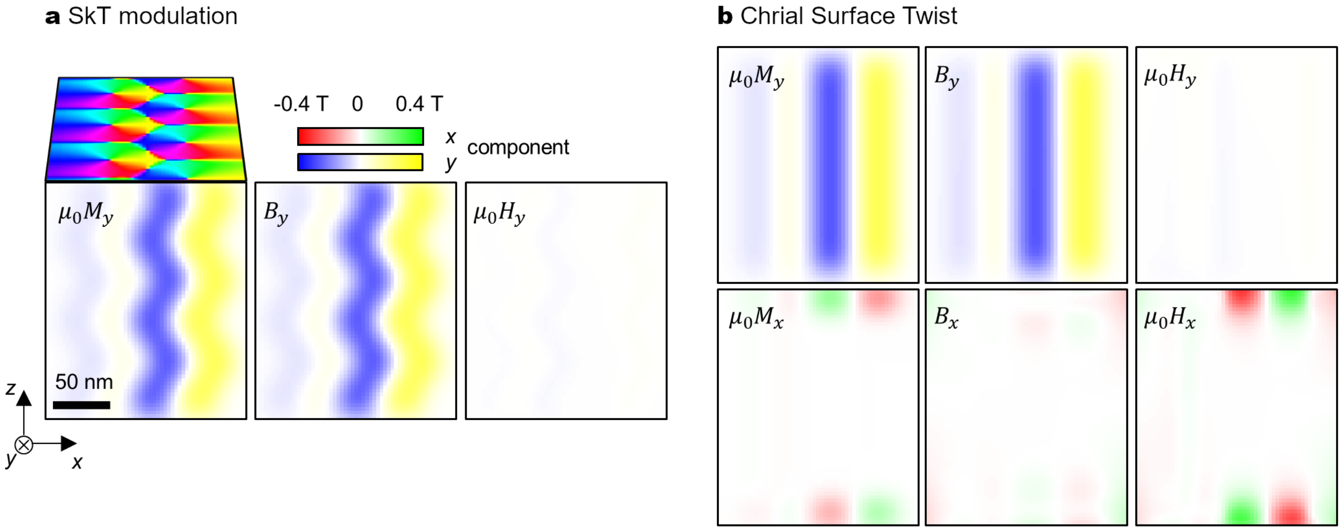

Figure S8: Idealized models of **a**, SkT modulation as a harmonic shift of skyrmion center and **b**, Néel modulations as a chiral surface twist starting 20 nm below each surface. The corresponding fields are depicted in the same color scheme employed in the main text, the top view of the needle in **a** contains a colormap of the in-plane rotation angle of $\boldsymbol{M}$.

SI 8 Skyrmion lattice packing

In order to analyze the packing of the skyrmions within the needle, the ratio of positive and negative B_z values inside a magnetic unit cell was calculated. To do so, the centres of the skyrmions for each z slice of the 3D stack were determined from the in-plane magnetic induction $B_\perp = \sqrt{B_x^2 + B_y^2}$. Connecting these points yields three magnetic unit cells within the field view (see red rhombi in the insets in Fig. S9). These unit cells were subsequently utilized to calculate the proportion of the areas, where B_z is pointing upward and downward. The resulting values for each z slice of the three unit cells are plotted in Fig. S9. The graphs show a non-trivial behaviour as function of the z position, which can be partly attributed to reconstruction artifacts of B_z. Nevertheless all three graphs manifest a ratio above 1 for most of the slices. Taking into account the limited number of samplings a mean value for the ratio of 1.5 was determined, indicating a closed packed skyrmion lattice[9].

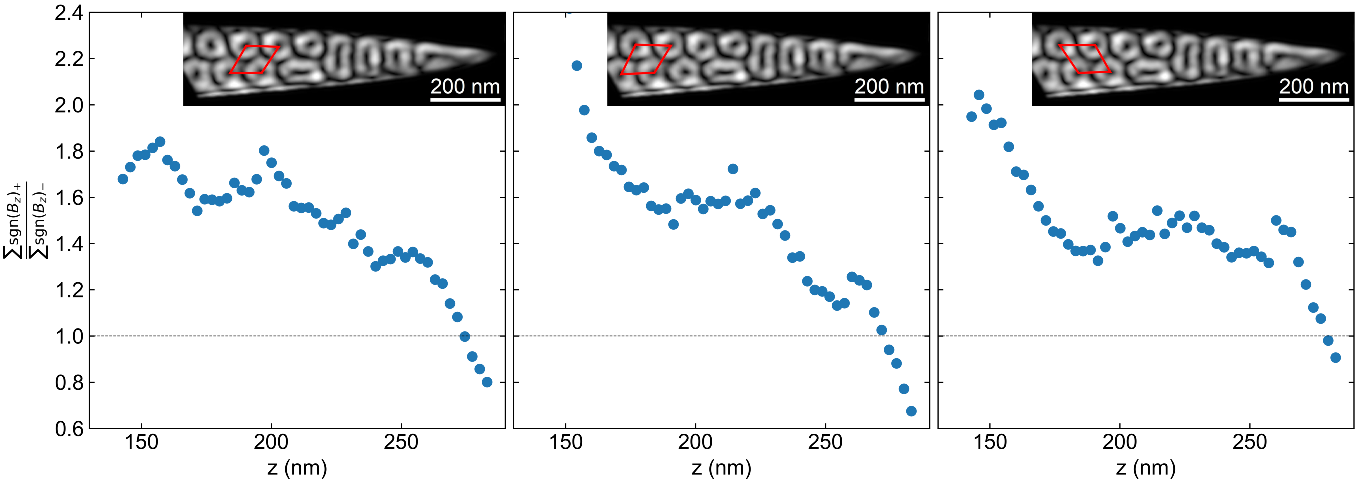

Figure S9: Proportion of positive and negative B_z values inside the magnetic unit cells (red rhombi in the insets) as function of the z position in the 3D stack.

SI 9 Magnetic energy densities

As already described in the main text, the representations of the energy densities (see Fig. 4 in the main text) were limited to the contributions dominated by the in-plane components of the magnetic induction due to persisting reconstruction artifacts in the B_z component and the general inability of VFET to reconstruct the magnetization directly (see also SI 7). In spite of this restriction it is possible to reveal the skyrmion energetics as shown in the following. The comparison of the energy terms calculated with the in-plane magnetic induction and total magnetization from magnetostatic simulations (see Fig. S10) confirms that the energetics derived from the in-plane components of $\boldsymbol{B}$ (equivalent to Fig. 4 in the main text) qualitatively agree with the full energy densities derived from $\boldsymbol{M}$. The increased exchange energy in the center and interstitial of the skyrmion is compensated by the DM energy density ultimately stabilizing the skyrmion.

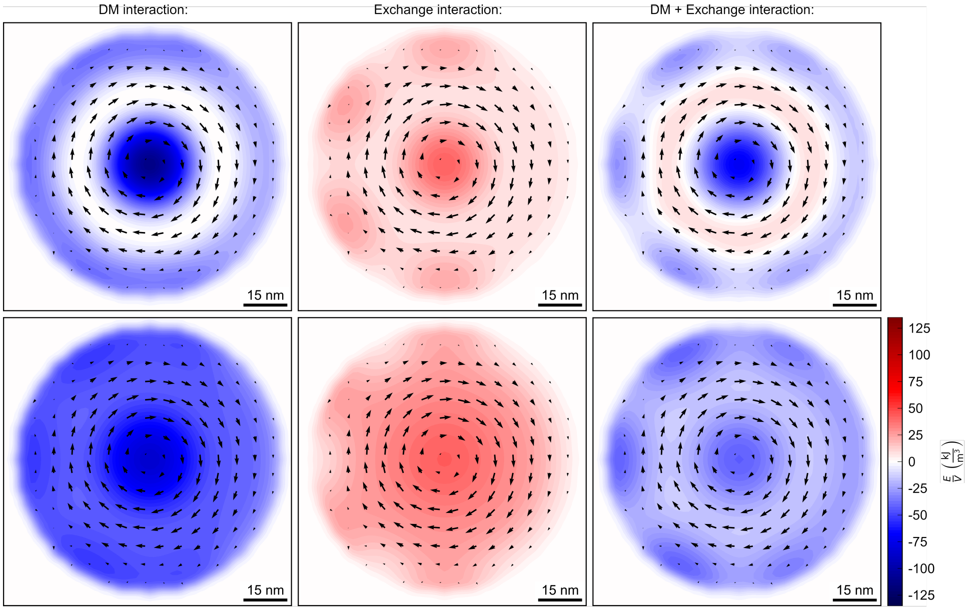

Figure S10: Top row: Representative magnetic energy density contributions $\frac{D}{\mu_0^2 M_s^2} B_z (\nabla \times \boldsymbol{B})_z$ (DM interaction), $\frac{A}{\mu_0^2 M_s^2} \left|(\nabla \times \boldsymbol{B})_z\right|^2$ (exchange interaction) and their sum from magnetostatic simulations. Bottom row: Corresponding energy contributions $\frac{D}{M_s^2} \boldsymbol{M} \cdot (\nabla \times \boldsymbol{M})$ and $\frac{A}{M_s^2} \left|\nabla \times \boldsymbol{M}\right|^2$ of the total magnetization from magnetostatic simulations. The arrows indicate the in-plane magnetic induction and magnetization respectively.

As displayed in Fig. S11 the restriction to the in-plane magnetic induction is consistently applicable to all skyrmions of the needle. There is a lowering of the total energy density in all skyrmion cores observable.

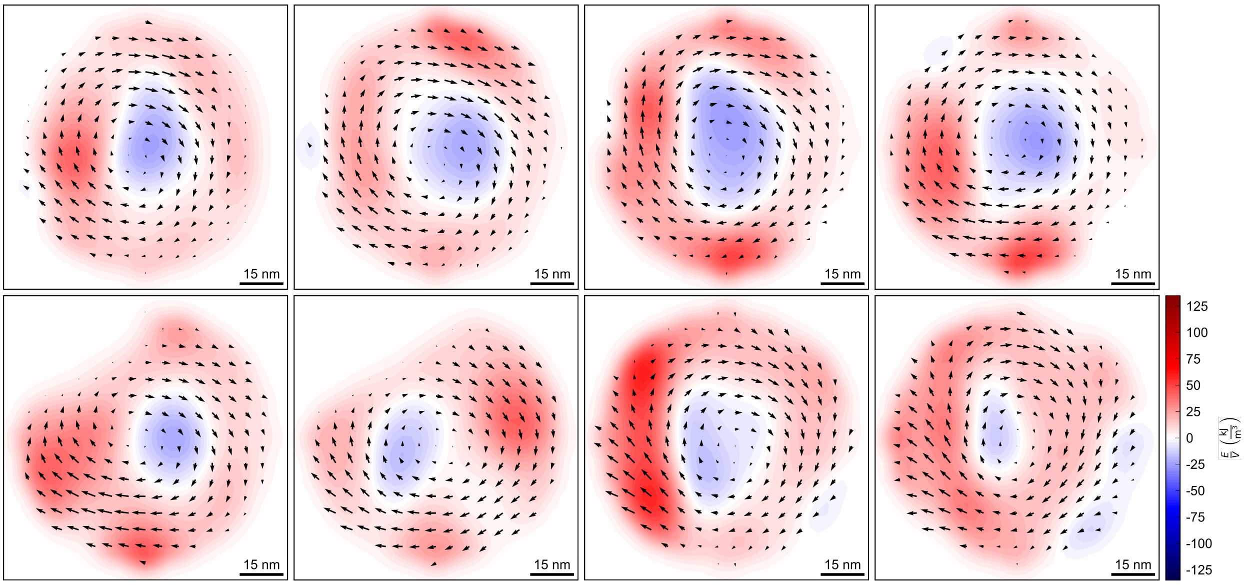

Figure S11: Sum of DM and exchange energy densities dominated by the experimental in-plane magnetic induction for all investigated skyrmions. The arrows indicate the in-plane magnetic induction.

SI 10 Hologram centerband and sidebands

Analyzing the Fourier transform of the electron wave, i.e., the holographic sideband visible in the Fourier transform of the electron hologram (see Fig. S12a) recorded in the thicker FeGe specimen region, we observe up to 3rd order systematic reflections of the magnetic skyrmion lattice (see Fig. S12b). They demonstrate the solitonic nature of the skyrmions, i.e., the arrangement of solitonic field modulations with a non-trivial structure factor in an ordered lattice. Alternatively, they may be also observed by investigating the rather pronounced non-harmonic rotation of the field when striding away from the center of a single skyrmion tube in the reconstructed 3D field. Fig. S12c shows an in-plane (x-y) slice through a skyrmion reconstructed by VFET (see Fig. 2 in the main text). The arrow (parallel to the y direction) indicates, where the line profile of the B_x component Fig. S12d is taken. It shows a larger slope of the modulation close to the skyrmion core that agrees well to theoretical models of single skyrmions and highly resolved experimental measurements with spin-polarized STM [10].

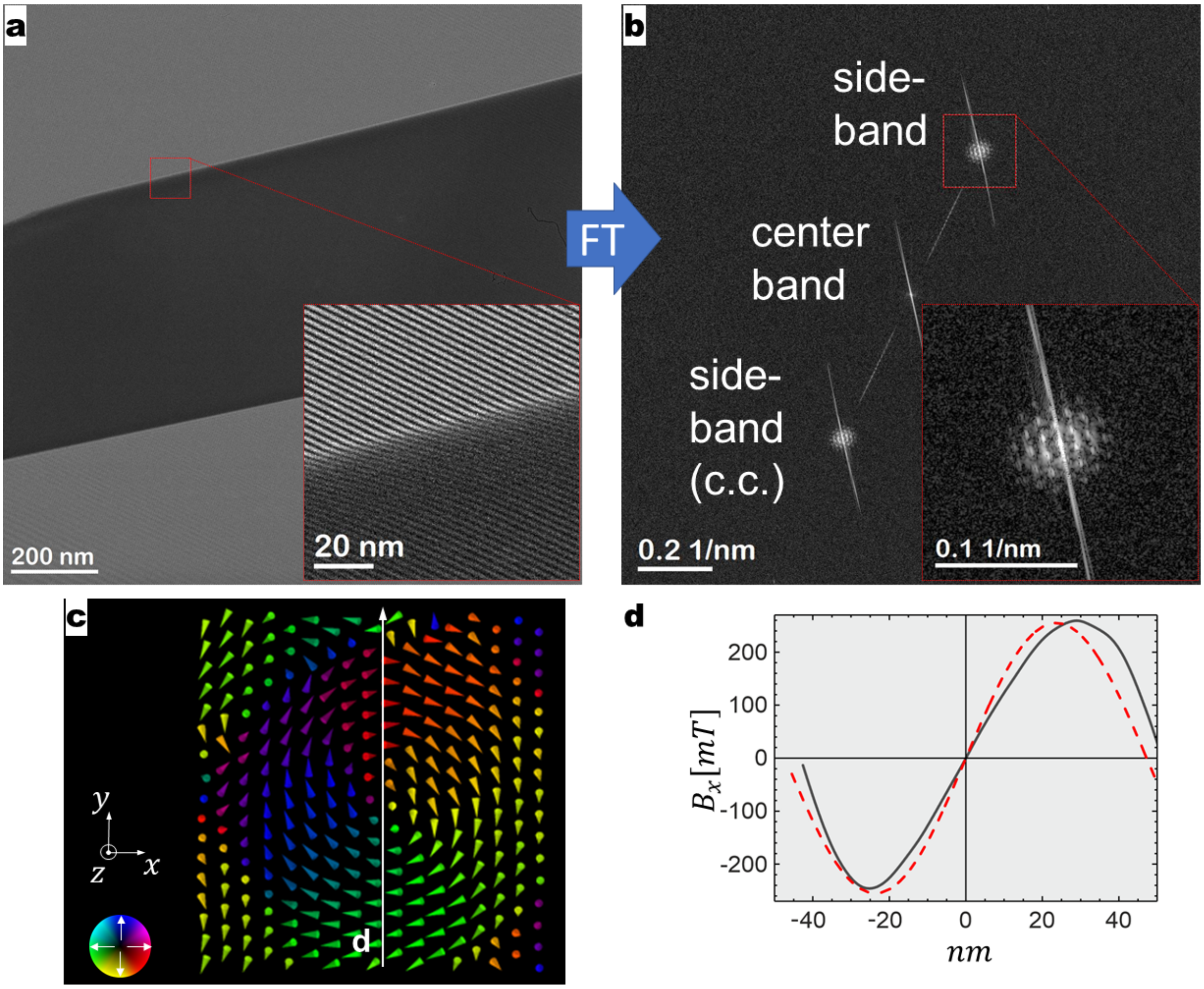

Figure S12: Observation of solitonic nature of skyrmions. **a**, Electron hologram of the FeGe needle-shaped FIB-prepared sample taken at 95 K at position shown in Fig. S1c. **b**, Fourier transform of **a** showing higher order reflections of the skyrmion lattice in holographic sideband (inset). **c**, Arrow plot of an in-plane (x-y) slice through a skyrmion reconstructed by VFET. **d**, Line profile of the B_x component taken along the line scan indicated by the white arrow in **c**.

SI 11 Electric and magnetic fringing fields

The FeGe needle specimen as well as the magnetic ring cause magneto-static fringing fields. In case the sample is slightly charged up by the electron beam, also electro-static stray fields may occur. If these far-reaching fields are too strong, they may disturb the tomographic reconstruction. Here, we demonstrate that these fields are small enough to not influence significantly the 3D reconstruction at the applied regularization. Fringing fields are visible in the vacuum regions of the phase images as phase modulation. Fig. S13 shows the cosine of 10× amplified phase images providing fringes that illustrate direction and strength of the projected in-plane components of the magnetic induction. In Fig. S13a, b, phase images at 0° tilt angle display similar density of the projected field lines in the vacuum for 95 K (skyrmion state) and room temperature (non-magnetic), hence the major contribution of the stray field comes from the magnetic ring. Consequently, after subtraction of both phase images and correction of a constant phase gradient, in the resulting magnetic phase image (see Fig. S13c) a smaller stray field is left. The same is true for phase images reconstructed at 45° (see Fig. S13d-f. Quantitative evaluation of the fringe distances in the magnetic phase images results in projected fields between 1.5 Tnm and 3.5 Tnm depending on tilt angle and position. Considering a back-projection path length of ca. 300 nm used for tomographic back-projection of the projection data, this corresponds to $\boldsymbol{B}$ field variations in the tomogram in the range of 0.01 T.

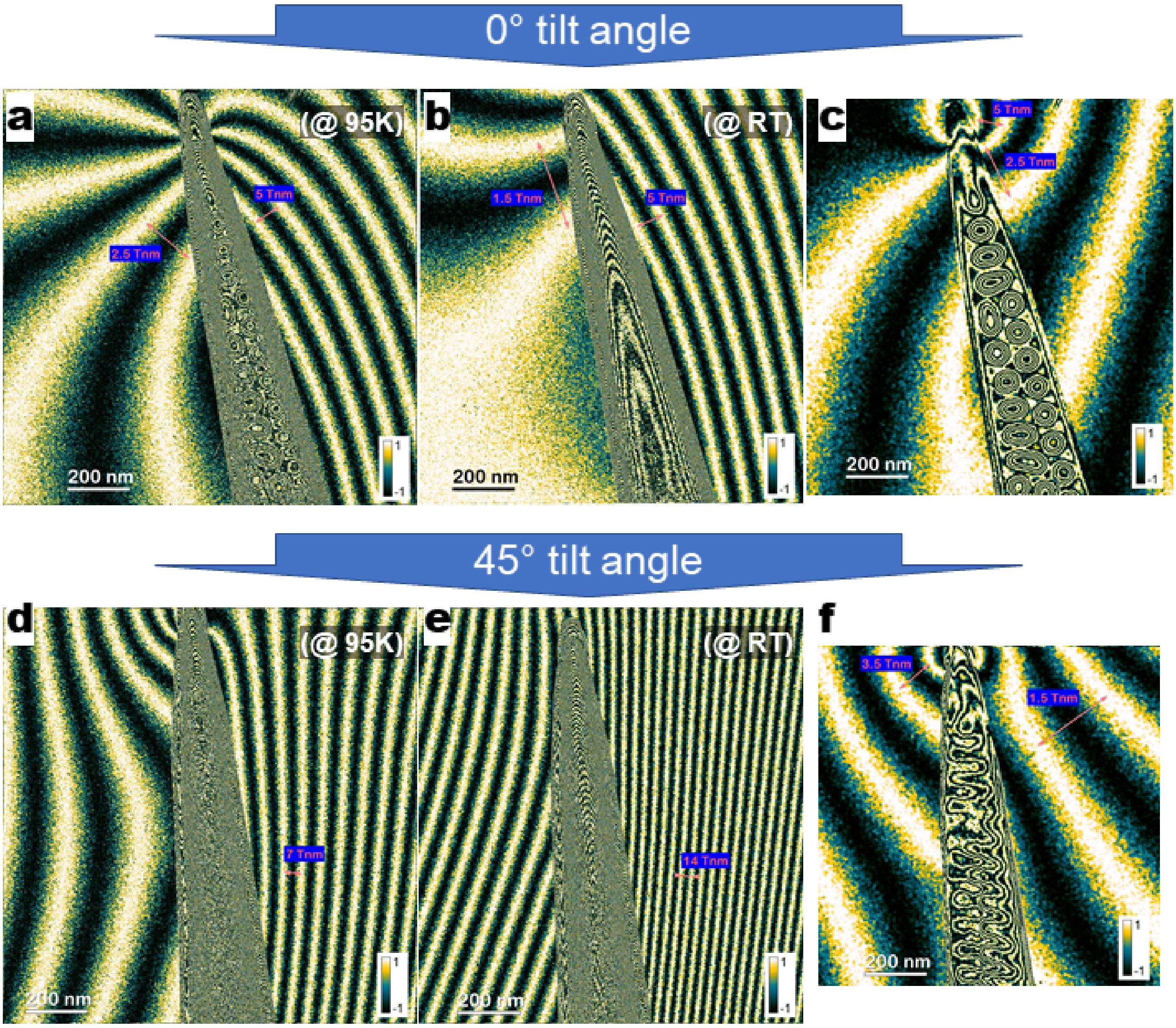

Figure S13: Fringing fields around the FeGe specimen observed in phase images. **a, b**, Cosine of phase images (10× amplified) illustrating the projected in-plane $\boldsymbol{B}$ field components taken at 0° and **a** 95 K, room temperature **b**. **c**, Magnetic phase image obtained by subtraction of **a** and **b**. **d-f**, Same as **a-c** but at 45° tilt angle.

SI 12 Tomographic reconstruction of simulated skyrmion needle

In order to verify the fidelity of the $\boldsymbol{B}$ field of the FeGe needle reconstructed in 3D from the experimental tilt series, we mimicked a tomographic vector-field reconstruction of a simulated magnetic skyrmion needle (see Fig. S14a) with similar dimensions, orientation, and sampling as in the experimental case (see Methods section of main text). In detail, we employed the following procedure:

1. Magnetostatic simulation of the entire 3D magnetic induction of the needle (see Sect. SI 7) as shown in Fig. S14a. The dimensions of the needle in x, y, and z direction are 200 nm by 750 nm by 200 nm. The orientation of the skyrmion tubes are along z direction.

2. Rotation of the simulated needle by 30° around z axis as in the experimental case.

3. Computation of the magnetostatic vector potential $\boldsymbol{A}$ by solving $-\Delta\boldsymbol{A} = \nabla \times \boldsymbol{B}$ (Coulomb gauge).

4. Simulation of the electrostatic scalar potential $V(x, y, z)$ using a constant mean inner potential (MIP) of 22 V within the needle.

5. Computation of the phase tilt series tilted about angles α around the y axis using the experimental tilt range. For each y position, the line integrals along d**s** depending on α and l the coordinate in the projection line read for the electrostatic part

$$\varphi_{el}\left(l, y, \alpha\right) = C_E \int_{-\infty}^{+\infty} V\left(x, y, z\right) \mathrm{d}\mathbf{s} \tag{2}$$

and for the magnetostatic part

$$\varphi_{mag}\left(l, y, \alpha\right) = -\frac{e}{\hbar} \int_{-\infty}^{+\infty} \left(A_x\left(x, y, z\right)\sin(\alpha) + A_z\left(x, y, z\right)\cos(\alpha)\right) \mathrm{d}\mathbf{s} \tag{3}$$

6. Rotation of the simulated needle by -60° around z axis and repetition of steps 3-5 to compute the second phase tilt series with the same parameters as in the experiment.

7. Calculation of phase tilt series $\varphi = \varphi_{el} + \varphi_{mag}$

8. Tomographic reconstruction of three 3D phase maps, two total ones rotated by 90° to each other, and an electrostatic one from the corresponding tilt series

9. Subtraction of the electrostatic 3D phase map from the other two 3D phase maps to obtain the magnetic 3D maps as described in the Methods part of the main text

10. Derivation of the two magnetic 3D phase maps in direction perpendicular to both the experimental tilt axis and tilt directions using an appropriate Fourier filter (Fourier-slice thoerem), as well as, multiplication with the factor $\hbar/e$ leading to the corresponding $\boldsymbol{B}$ field components.

11. Calculation of B_z from B_x and B_y as described in the Methods part of the main text.

The results are depicted in Fig. S14 visualized in a similar way as Fig. 2 of the main text. It clearly shows that all three $\boldsymbol{B}$ field components can be reconstructed in a remarkable quality using the here presented workflow applied to a tilt series with incomplete tilt range. The limitations of the tomograms are mainly noticeable by a reduced resolution in z direction (missing wedge artefacts), in particular visible in the B_z component (see Fig. S14a), but also in the other two components the elongation and gradual reduction of the signal along z direction (see Fig. S14b, c) is visible.

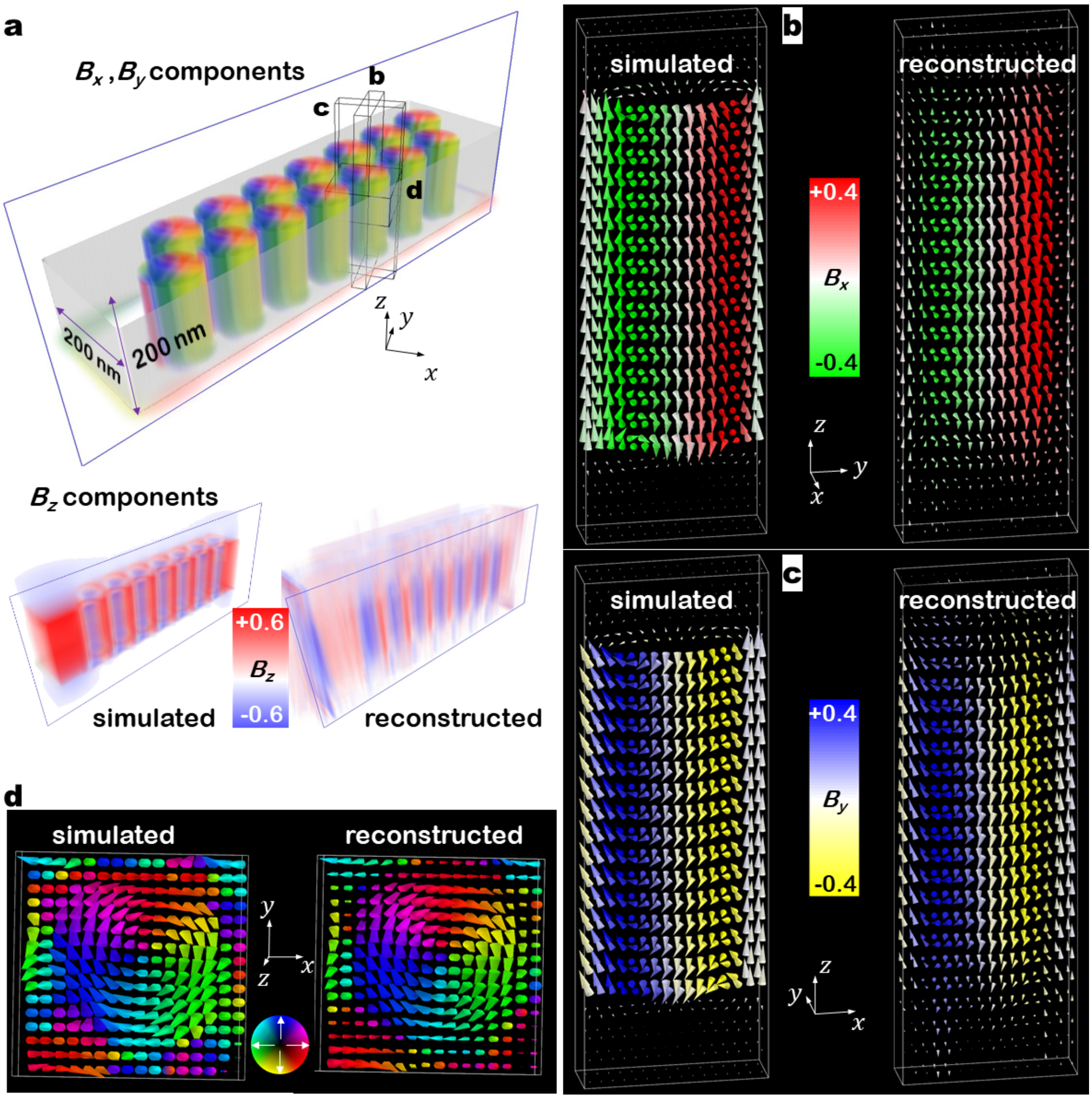

Figure S14: Comparison of a simulated skyrmion needle with its tomographic reconstruction using the same reconstruction workflow and parameters as in the experimental case. **a**, Color-coded volume rendering of simulated B_x (green-red) and B_y (yellow-blue) components. Likewise to the experimental case (see Fig. S1), the needle-axis is rotated 30° to the x axis. The simulated and reconstructed B_z components are volume-rendered in blue-red. The elongation (blurring) of the B_z reconstruction along z direction due to the incomplete tilt range is clearly visible. **b**, According to Fig. 2 of the main text, 2D cross sections along **b** the y-z, **c**, the x-z, and **d**, the x-y planes as indicated in **a** are taken for both cases.

References

[1] Gutfleisch, O. High-temperature samarium cobalt permanent magnets. In *Nanoscale Magnetic Materials and Applications*, 337–372 (Springer US, 2009).

[2] Chen, C. H., Walmer, M. H., Kottcamp, E. H. & Gong, W. Surface reaction and Sm depletion at 550 °C for high temperature Sm-Tm magnets. *IEEE Transactions on Magnetics* **37**, 2531–2533 (2001).

[3] Schneider, S. *et al.* Induction Mapping of the 3D-Modulated Spin Texture of Skyrmions in Thin Helimagnets. *Physical Review Letters* **120**, 217201 (2018).

[4] Wolf, D. *et al.* Three-Dimensional Composition and Electric Potential Mapping of III-V Core-Multishell Nanowires by Correlative STEM and Holographic Tomography. *Nano Letters* **18**, 4777–4784 (2018).

[5] Wolf, D. *et al.* Holographic vector field electron tomography of three-dimensional nanomagnets. *Communications Physics* **2**, 1–9 (2019).

[6] Bogdanov, A. & Hubert, A. Thermodynamically stable magnetic vortex states in magnetic crystals. *Journal of Magnetism and Magnetic Materials* **138**, 255–269 (1994).

[7] Beg, M. *et al.* Ground state search, hysteretic behaviour, and reversal mechanism of skyrmionic textures in confined helimagnetic nanostructures. *Scientific Reports* **5**, 1–14 (2015).

[8] Rybakov, F. N., Borisov, A. B. & Bogdanov, A. N. Three-dimensional skyrmion states in thin films of cubic helimagnets. *Physical Review B* **87**, 1–4 (2012).

[9] Balkind, E., Isidori, A. & Eschrig, M. Magnetic skyrmion lattice by the Fourier transform method. *Physical Review B* **99**, 134446 (2019).

[10] Leonov, A. O. *et al.* The properties of isolated chiral skyrmions in thin magnetic films. *New Journal of Physics* **18**, 065003 (2016).
